# Systemic Rotenone Administration Causes Extra-Nigral Alterations in C57BL/6 Mice

**DOI:** 10.3390/biomedicines10123174

**Published:** 2022-12-07

**Authors:** Sarah Thomas Broome, Alessandro Castorina

**Affiliations:** Laboratory of Cellular and Molecular Neuroscience, School of Life Sciences, Faculty of Science, University of Technology Sydney, Sydney 2007, Australia

**Keywords:** neurotoxicity, rotenone, extranigral toxicity, Parkinson’s disease, central nervous system, neuroinflammation, neuropeptides

## Abstract

Systemic administration of rotenone replicates several pathogenic and behavioural features of Parkinson’s disease (PD), some of which cannot be explained by deficits of the nigrostriatal pathway. In this study, we provide a comprehensive analysis of several neurochemical alterations triggered by systemic rotenone administration in the CNS of C57BL/6 mice. Mice injected with either 1, 3 or 10 mg/kg rotenone daily via intraperitoneal route for 21 days were assessed weekly for changes in locomotor and exploratory behaviour. Rotenone treatment caused significant locomotor and exploratory impairment at dosages of 3 or 10 mg/kg. Molecular analyses showed reductions of both TH and DAT expression in the midbrain, striatum and spinal cord, accompanied by altered expression of dopamine receptors and brain-derived neurotrophic factor (BDNF). Rotenone also triggered midbrain-restricted inflammatory responses with heightened expression of glial markers, which was not seen in extra-nigral regions. However, widespread alterations of mitochondrial function and increased signatures of oxidative stress were identified in both nigral and extra-nigral regions, along with disruptions of neuroprotective peptides, such as pituitary adenylate cyclase-activating polypeptide (PACAP), vasoactive intestinal peptide (VIP) and activity-dependent neuroprotective protein (ADNP). Altogether, this study shows that systemic rotenone intoxication, similarly to PD, causes a series of neurochemical alterations that extend at multiple CNS levels, reinforcing the suitability of this pre-clinical model for the study extra-nigral defects of PD.

## 1. Introduction

Parkinson’s disease (PD) is a progressive neurodegenerative disorder that affects around 2% of the population over the age of 60 [1,2]. It clinically manifests with motor impairments, including bradykinesia, tremor, rigidity and postural instability [3]. These classical parkinsonian motor symptoms are caused by the degeneration of dopamine neurons in the substantia nigra pars compacta (SNpc), resulting in a lack of dopamine in the striatum [4]. Notwithstanding these pathological hallmarks, PD patients also develop a myriad of non-motor symptoms, including depression, anxiety, cognitive deficits and autonomic dysfunction, suggesting that damage might also occur in other regions of the central nervous system (CNS) [5]. 

The driving force triggering the neurodegeneration observed in PD remains unknown; however, several factors contribute to disease pathogenesis, including genetics, environmental factors, oxidative stress and neuroinflammation [6]. 

Rotenone is a naturally occurring compound used commercially as a pesticide and piscicide, which reproduces some of the major clinical and behavioural features of PD in rodents [7,8,9]. Since its discovery as a PD-mimetic, the rotenone intoxication model has become a popular preclinical model of PD based on the high lipophilic nature of the compound, which enables it to cross biological membranes allowing systemic administration, but also due to its ability to trigger several pathological mechanisms, including oxidative stress, protein aggregation and CNS inflammation, as well as its capability of reproducing a PD-like behavioural phenotype [10]. This ability to reproduce key pathological and phenotypic features of PD has made rotenone a valuable tool to study drug-mediated neuroprotection [11].

Rotenone administered at low dosages induces degeneration of the dopaminergic nigrostriatal pathway and causes motor deficits [12,13,14,15,16]. Rotenone-mediated dopaminergic degeneration occurs via the inhibition of the mitochondria electron transport chain complex I, which results in the formation and accumulation of reactive oxygen species, leading to oxidative stress [9,17]. Studies of human *post-mortem* brain tissues indicate that oxidative stress and accumulation of reactive oxygen species are critical events in the pathogenesis of PD [18,19].

Chronic daily intraperitoneal injections of rotenone induce l-3,4-dihydroxyphenylalanine (L-DOPA) responsive locomotor deficits and neurochemical abnormalities characteristic of PD in both rats and mice [15,16,20,21]. However, most of these investigations have been performed using non-standardised rotenone injection regimens, which often produce a certain degree of variability–mainly depending on the route of administration, concentration, animal strain and experimental regimes, making it difficult to compare studies [22]. Additionally, most of these investigations focus on the analyses of neurochemical alterations in the midbrain and striatum (i.e., the nigrostriatal pathway), partly overlooking for any possible neurochemical changes in other regions of the CNS [23]. 

Based on the evidence indicating the existence of common clinical and pathological features shared by the rotenone toxicity model and PD patients, in the present study we aimed at further characterising the range of neurochemical alterations triggered by systemic rotenone administration in the CNS of C57BL/6 mice. Once we established the suitability of the animal model by behavioural assessments, we determined if rotenone toxicity disrupted the expression of dopamine, oxidative stress and inflammatory markers, as well as neuropeptides and trophic factors in at least six distinct CNS sites. Our results indicate that rotenone triggers widespread neurochemical changes that extend beyond the nigrostriatal dopaminergic system. Altogether, these findings suggest that the neurodegenerative process triggered by rotenone intoxication, similarly to PD, affects multiple central and perhaps peripheral systems, offering a viable model to investigate both nigral and extra-nigral defects triggered by PD.

## 2. Materials and Methods

### 2.1. Animal Experiments

Twenty-four 7-week-old male C57BL/6 mice were purchased from ARC (Perth, WA, Australia). Mice were allowed to acclimate for one-week and experimental regimen commenced when mice were 8 weeks old. Mice were housed in individually ventilated cages (4 mice per cage), under normal 12:12 h light/dark cycle, with access to food and water *ad libitum.* All experiments were conducted in line with the Australian Code of Practice for the Care and Use of Animals for Scientific Purposes, and in compliance with the ARRIVE guidelines. The University of Technology Sydney Animal Care and Ethics Committee (ETH19-3322) approved all animal experiments. 

### 2.2. Rotenone Experimental Protocol

Mice were randomly assigned to one of the four treatment groups: control (vehicle), rotenone 1 mg/kg, 3 mg/kg or 10 mg/kg. Rotenone was prepared as a stock solution in 0.1% DMSO diluted in 0.1% saline. Mice underwent behaviour testing every 7 days to assess locomotor and exploratory behaviour. Mice in each group were daily injected with intraperitoneal injections as indicated for 21 days and monitored for 2 h post injection. On day 22, mice were sacrificed and brains and spinal cord were collected. Tissue was snap frozen and used to perform molecular analysis. The brains were subsequently micro dissected into the following regions: prefrontal cortex, striatum, hippocampus, amygdala and midbrain with one hemisphere used for RNA analyses and the other for protein analyses. 

### 2.3. Open Field Behaviour Test

The open field (OF) test was conducted every 7 days in the light cycle between 08:00 h and 12:00 h. Animals were acclimated in the testing rooms for 30 min for habituation. The OF was conducted in the dark. The OF consisted of a square box (40 × 40 cm ) and 50 cm tall made of grey plexiglass. Mice were placed individually in the centre of the area and allowed to explore the environment freely for 5 min while being recorded. The OF was cleaned thoroughly between each mouse to eliminate any odour cues with 70% ethanol. The FIJI/ImageJ Plugin MouBeAt software [24] was used to analyse videos. MouBeAt quantifies the distance and time spent in the central and peripheral areas, number of entries to centre region and average speed. An entry into an area was counted when at least 70% of the mouse body had completely entered the area. For this type of analyses, data was computed using a repeated measures 2-way ANOVA, factoring both treatment and time as independent variables.

### 2.4. Real-Time Quantitative Polymerase Chain Reaction (RT-qPCR)

Briefly, total RNA was extracted using TRI-reagent (Sigma-Aldrich, Castle Hill, NSW, Australia) and chloroform and precipitated with ice-cold 2-propanol following established protocols [25,26]. RNA concentration was determined using spectrophotometry (Nanodrop ND-1000^®^ spectrophotometer, Wilmington, DE, USA). Single-stranded cDNA was synthesized using Tetro cDNA synthesis kit (Bioline, Sydney, NSW, Australia). Real-time qPCR was performed to analyse the steady-state mRNA levels of twelve genes (Table 1). The ribosomal protein 18S was used as the housekeeping gene. Each reaction consisted of 3 μL cDNA (final concentration 100 ng), 5 μL iTaq Universal SYBR Green Master Mix (BioRad, VIC, Australia), 0.8 μL of forward and reverse primers. To examine changes in expression, the mean fold changes of each sample was calculated using the ΔΔCt method as previously described [27,28]. PCR product specificity was evaluated by melting curve analysis, with each gene showing a single peak (data not shown). 

### 2.5. Protein Extraction and Western Blot

Protein was extracted by homogenizing tissues in radioimmunoprecipitation assay (RIPA) buffer containing a protease inhibitor to preserve protein integrity (cOmplete^TM^, Mini, EDTA-free Protease Inhibitor Cocktail, Sigma-Aldrich, Castle Hill, NSW, Australia) [29]. Tissues were sonicated and then cleared by centrifugation at 12,000× *g* for 10 min. Protein quantification was determined with bicinchoninic acid assay (Pierce BCA Protein Assay Kit, ThermoFisher Scientific, North Ryde, NSW, Australia). Equal amounts of protein (30 μg) was separated by SDS-polyacrylamide gel electrophoresis (SDS-PAGE) using 4–20% Mini-PROTEAN TGX Stain-Free Gels (15 well, BioRad, South Granville, NSW, Australia). The Precision Plus Protein Prestained Standard in All Blue (BioRad, South Granville, NSW, Australia) was included for comparison. Transfer to a PVDF membrane was performed using the semi-dry method (BioRad Trans-Blot Turbo Transfer System). Incubation with primary antibodies was performed overnight in 5% skim milk in TBST blocking solution at 4 °C. Antibodies and dilutions are summarized in Table 2. Western blots were visualized using chemiluminescence BioRad Clarity Western ECL Blotting Substrate Solution. Images were acquired using the BioRad ChemiDoc MP System. Images were analysed using Fiji ImageJ and ratios normalized to GAPDH which was used as a loading control. When possible, membranes pertaining to the same experimental group were stripped using a mild stripping buffer (1.5% [*w*/*v*] glycine, 1% [*w*/*v*] SDS, and 1% (*v*/*v*) Tween-20 in milliQ H20, pH 2.2) and re-incubated with GAPDH for quantification. Corresponding full blots are available in the Supplementary Material.

### 2.6. Immunohistochemistry

Brains were fixed in 4% paraformaldehyde for 48 h before dehydration and embedding in paraffin. 5 μm thick coronal sections were cut with using a microtome and mounted on glass slides. The sections were deparaffinised in xylene and rehydrated through decreasing concentrations of ethanol. Dopaminergic neurons were labeled with tyrosine hydroxylase (TH) (1:500, ab112, Abcam). The immunoreactivity of the antibody was revealed using the Rabbit specific HRP/DAB (ABC) Detection IHC Kit (ab64261, Abcam, VIC, Australia) according to manufacturer’s protocol. Hematoxylin was used to counterstain nuclei and to appreciate the gross architecture of the examined CNS region. Sections were dehydrated in increasing concentrations of ethanol and xylene before being mounted. Images were taken on a ZEISS AxioScan.Z1 at ×20 magnification. ImageJ software was used to quantify TH-staining intensity in the region of interest [30]. 

### 2.7. Statistical Analysis

All data are reported as mean ± S.E.M. Statistical analyses were performed using GraphPad Prism software ver. 9.0.2. (GraphPad Software, La Jolla, CA, USA). For behavioural analyses, in order to determine a possible relationship between treatment (drug dosage) and duration, two-way repeated measures ANOVA followed by Tukey post hoc test was performed, factoring both treatment regime and time as independent variables. All the other comparisons involving only two groups were analysed by unpaired Student’s t-test. Comparisons between multiple groups were analysed in function of a single independent variable (rotenone treatment), where results attained from mice receiving increasing dosages of rotenone were compared with vehicle-treated mice. Therefore, unless otherwise specified, these data were analyzed by one-way ANOVA followed by Dunnett’s post hoc test. All *p*-values ≤ 0.05 were considered statistically significant.

## 3. Results

### 3.1. Rotenone Intoxication Impairs Locomotion and Exploratory Behaviour in Mice

**Figure 1:**
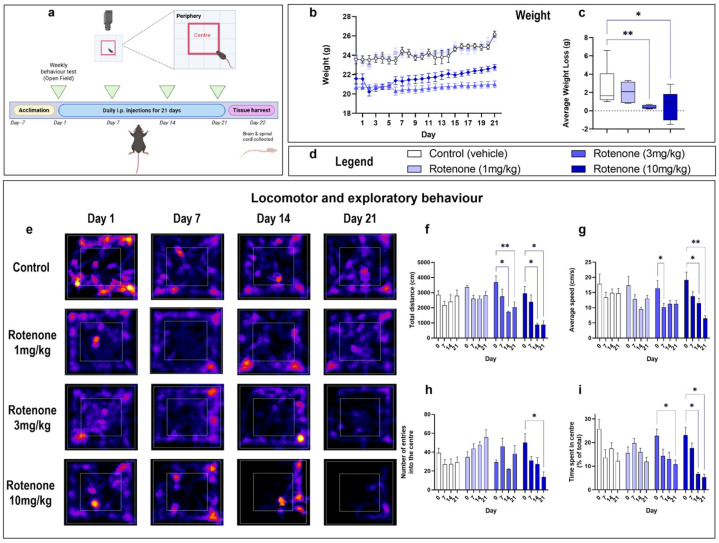
Rotenone impairs locomotor and exploratory behaviour. Experimental timeline for injections and behavioural assessments (**a**). The Open Field Test mice was used to assess locomotor and exploratory behaviour in rotenone-treated mice vs. baseline measurements every 7 days. The centre quadrant was defined as a central square with a surface area that is 25% smaller than the total area (red box), whereas the peripheral area was defined as the surface area between the centre quadrant and the walls of the Open Field box. Mice received an intraperitoneal injection of the indicated treatment daily for 21 days. On day 22, mice were humanely sacrificed and the brain and spinal cord were collected. Mean daily weight per treatment group (**b**) and the average weight loss/gain (**c**) were calculated using mean daily weights of day 1 vs. day 22. Corresponding legends of experimental treatments are shown in (**d**). Representative heat maps from MouBeAt Software that track the movement of mice during the open field test (**e**). Locomotor and behavioural measurements were determined by MouBeAt software. Heat maps use colour to represent time spent in a given area, ranging from blue (short duration) to red/yellow (long duration). Comparisons were made within the same treatment group compared to baseline measurements. An entry into an area was counted when at least 7% of the mouse body had completely entered the area. Total distance travelled (cm) in 5 min (**f**). Average speed reported as cm/s (**g**). Number of entries in centre (**h**). Time spent in centre (s) (**i**). Data are shown as means of n = 6–10 mice per group. * *p* < 0.05 or ** *p* < 0.01 as determined by one-way ANOVA followed by Dunnett’s post hoc test (weight change) or two-way repeated measures ANOVA followed by Tukey post hoc test (behavioural measurements).

To assess if rotenone induced changes in locomotor and/or exploratory behaviour, mice were subjected to the Open Field (OF) test as outlined in the experimental design (Figure 1a).

Mice were weighed daily prior to injection (Figure 1b). Analyses of body weight revealed that mice receiving 3 mg/kg and 10 mg/kg of rotenone gained significantly less weight compared with saline-injected controls (** *p* = 0.01 and * *p* = 0.0247 vs. saline, respectively; Figure 1c). 

MouBeAt, an ImageJ plugin, was used to track and quantify general locomotor (i.e., average speed and total distance travelled) and exploratory behaviours of mice in the OF (i.e., number of entries and total time spent in the centre quadrant) [24], which allowed us to generate representative heat maps depicting the locomotor pattern of tested mice (Figure 1d). Drug-induced deficits in locomotor activity were assessed by comparing the total distance travelled (F (9, 6) = 2.723, ** *p* = 0.0099; Figure 1e) and average speed (F (9, 60) = 1.631, *p* = 0.1269; Figure 1f) at 7, 14 and 21 days with respect to baseline-measurements taken prior to exposure to rotenone. To examine exploratory behaviour, we measured the number of times (F (9, 60) = 3.648, ** *p* = 0.0011; Figure 1g) and the total time spent by each mouse in the centre quadrant (F (9, 60) = 3.062, ** *p* = 0.0044; Figure 1h) of the OF. Mice display a natural aversion to open areas and preferentially stay close to the walls of the field (thigmotaxis). In contrast, they display an instinctive drive to explore a perceived threatening environment [31]. Therefore, the overall number of times a mouse enters the centre quadrant and the total time spent with respect to the periphery are indicative of the exploratory behaviour of mice (Figure 1a). 

We report that only mice administered with 3 mg/kg or 10 mg/kg of rotenone developed deficits in locomotor and exploratory behaviours as compared with baseline controls. In more detail, at 3 mg/kg, rotenone significantly reduced the total distance travelled in the OF on day 14 (* *p* = 0.0207), and this was further reduced by day 21 (** *p* = 0.0028) (Figure 1e), although this was not associated with an overall reduction in speed, except on day 7 (* *p* = 0.0442). In contrast, at this rotenone dosage, reductions in exploratory behaviour were only recorded on day 21 (* *p* = 0.0398; Figure 1h). 

As expected, at the highest rotenone dosage tested in this study (10 mg/kg), the drug severely impaired both locomotor and exploratory behaviours already after 14 days of rotenone treatment. Specifically, there was a significant reduction in the total distance travelled by these mice at both days 14 (* *p* = 0.0214 vs. baseline) and 21 (* *p* = 0.0207 vs. baseline; * *p* = 0.0201 vs. Day 7) (Figure 1e). This was correlated with significantly slower average speed at days 14 (* *p* = 0.0160 vs. baseline) and 21 (** *p* = 0.0093 vs. baseline; * *p* = 0.0324 vs. Day 7) (Figure 1f). Additionally, mice exhibited reduced exploratory behaviour, as demonstrated by the reduction in the number of entries in the centre of the OF at day 21 (* *p* = 0.0458) (Figure 1g) and the reduction in the time spent in the centre vs. the periphery, recorded both at day 14 (* *p* = 0.0317 vs. baseline; ** *p* = 0.0094 vs. Day 7) and at day 21 (* *p* = 0.0342 vs. baseline; * *p* = 0.0111 vs. Day 7) (Figure 1h).

### 3.2. Rotenone Reduces the Expression of Dopaminergic Markers in the Midbrain, Striatum and Spinal Cord

**Figure 2:**
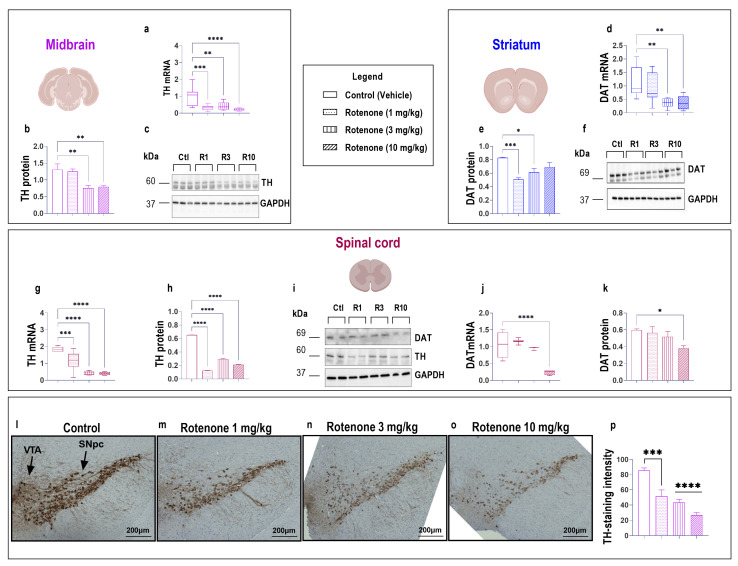
Rotenone reduces the expression of dopaminergic markers in the midbrain, striatum and spinal cord. On day 22, mice from vehicle- and rotenone-treated groups (1, 3 and 10 mg/kg) were sacrificed and midbrains, striata and spinal cords were collected for molecular analyses. TH mRNA and protein expression in the midbrain (**a**–**c**). DAT mRNA and protein expression in the striatum (**d**–**f**). TH and DAT mRNA and protein expression in the spinal cord (**g**–**k**). Representative photomicrographs showing TH-immunoreactivity and semi-quantitative measurement of staining intensity in the midbrain of vehicle-, 1, 3 and 10 mg/kg rotenone-treated mice (**l**–**p**). At least two sections from n = 3 mice per group were used for semi-quantitative analyses of staining intensity. Scale bar = 200 μm. Gene expression was measured using real-time qPCR and quantified using the ΔΔCt method after normalization to s18 (ribosomal protein s18 gene), the housekeeping gene. Real-time qPCR results are presented as mean fold changes with respect to vehicle-treated mice (control). In Western blots, protein expression was normalized to GAPDH, used as the loading control. Densitometry results are expressed as mean ± S.E.M. Data in qPCR, Western blots and immunohistochemistry represents means of n = 4 samples for each group. * *p* < 0.05, ** *p* < 0.01, *** *p* < 0.001 or **** *p* < 0.0001 as determined by one-way ANOVA followed by Dunnett’s post hoc test. Ctl: control; R1, 3, 10: Rotenone (1, 3, 10 mg/kg); TH: tyrosine hydroxylase; DAT: dopamine transporter; GAPDH: glyceraldehyde 3-phosphate dehydrogenase; kDa: Kilodalton; VTA: ventral tegmental area; SNpc: Substantia Nigra pars compacta.

PD is characterized by the loss of dopamine neurons in the midbrain, which results in a loss of dopamine availability in the *striatum* [32]. In addition, there is evidence that tyrosine hydroxylase positive (TH)^+^ and dopamine receptors’-expressing neurons are also localized in the spinal cord [33,34], implicating a role of the spinal dopaminergic system in motor control. As such, we sought to analyse the expression of two main dopamine markers, TH and dopamine transporter (DAT), in the midbrain, striatum and spinal cord of mice to determine if rotenone caused a similar loss of dopaminergic neurons throughout the CNS (Figure 2). 

In the midbrain, mice that received either 1 mg/kg, 3 mg/kg or 10 mg/kg of rotenone displayed a significant reduction in TH mRNA expression (*** *p* = 0.0002, ** *p* = 0.0013 and **** *p* < 0.0001 vs. vehicle, respectively; F (3, 32) = 11.07; Figure 2a). Similarly, protein expression studies confirmed the significant reduction of TH expression, but only at 3 mg/kg and 10 mg/kg (** *p* = 0.0045 or ** *p* = 0.0019 vs. vehicle, respectively; F (3, 15) = 9.902; Figure 2b,c). 

Striatal DAT transcript levels were significantly reduced at 3 mg/kg (** *p* = 0.0043 vs. vehicle) and 10 mg/kg rotenone (** *p* = 0.0049; F (3, 28) = 6.262; Figure 2d). These results were paralleled by a decline of DAT protein expression in mice administered with 1 mg/kg (*** *p* = 0.0003 vs. vehicle) or 3 mg/kg (* *p* = 0.0107), but surprisingly not in animals treated with 10 mg/kg rotenone (*p* > 0.05; F (3, 20) = 8.527; Figure 2e,f). 

The expression of dopamine markers in the spinal cord revealed a significant reduction of TH transcripts across all treatment groups (**** *p* < 0.0001; F (3,28) = 41.55; Figure 2g), including rotenone 1 mg/kg (*** *p* = 0.0002 vs. vehicle), with both higher doses–3 mg/kg and 10 mg/kg–able to further reduce TH transcript levels (**** *p* < 0.0001; Figure 2g). These results were paralleled by similar and significant reductions of TH protein expression at all rotenone doses tested (**** *p* < 0.0001; F (3, 20) = 1282; Figure 2h,i). Analyses of DAT mRNAs in the spinal cord did not show changes until the highest rotenone dosage (10 mg/kg), where a significant reduction was observed (**** *p* < 0.0001 vs. vehicle; F (3, 28) = 40.88; Figure 2j). Similarly, DAT protein levels were only reduced at the highest rotenone dosage (* *p* = 0.0172; F (3, 20) = 3.566; Figure 2i,k).

Our rationale to test multiple dosages of the insecticide was to identify a dosage that reliably induced PD-like pathophysiology and associated behavioural deficits. Both behaviour (Figure 1) and dopaminergic markers (Figure 2) confirmed that 10 mg/kg rotenone reliably reproduced the pathological and clinical features of PD. These data were further supported by immunohistochemical evidence of reduced TH^+^ staining in the midbrain of mice exposed to increasing dosages of rotenone compared with controls (*** *p* < 0.001 [1 mg/kg rotenone vs. vehicle], **** *p* < 0.0001 [3 and 10 mg/kg rotenone vs. vehicle, respectively]; F (3, 20) = 21.82; Figure 2l–p).

### 3.3. Rotenone Triggers CNS Region-Specific Changes in the Expression of Dopamine Receptors and Brain-Derived Neurotrophic Factor

Dopamine is the main neurotransmitter affected in PD pathogenesis as a result of the loss of dopaminergic neurons. To examine potential disturbances of the dopaminergic system in nigral and extra-nigral regions, we analysed the mRNA expression of all dopamine receptors both in vehicle- and 10 mg/kg rotenone-treated mice. 

**Figure 3:**
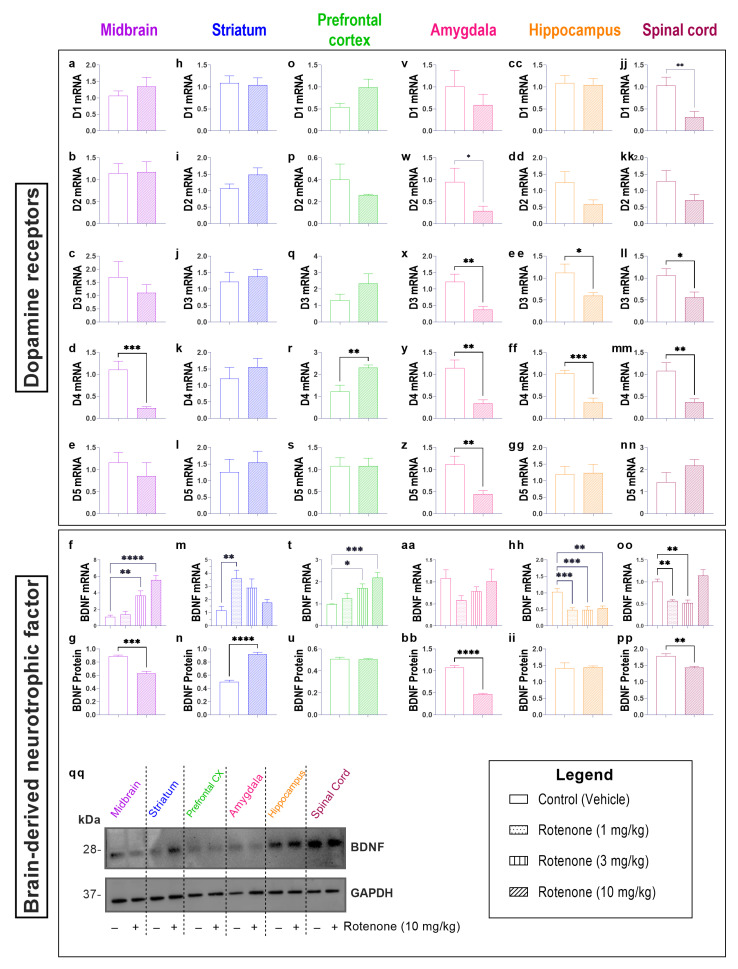
Rotenone disturbs the expression of dopamine receptors and brain-derived neurotrophic factor in specific CNS-regions. On day 22, mice from vehicle- and rotenone-treated groups (10 mg/kg) were sacrificed and midbrains, striata, prefrontal cortices, amygdala, hippocampi and spinal cords were collected for real-time qPCR analyses. mRNA expression of the five dopamine receptors (D1–5) in the midbrain (**a**–**e**), striatum (**h**–**l**), prefrontal cortex (**o**–**s**), amygdala (**v**–**z**), hippocampus (**cc**–**gg**) and spinal cord (**jj**–**nn**). BDNF mRNA expression and protein expression, including representative Western blots (**qq**) was analysed in the midbrain (**f**,**g**), striatum (**m**,**n**), prefrontal cortex (**t**,**u**), amygdala (**aa,bb**), hippocampus (**hh**,**ii**) and spinal cord (**oo**,**pp**). Gene expression was measured using real-time qPCR and quantified using the ΔΔCt method after normalization to s18 (ribosomal protein s18 gene), the housekeeping gene. Real-time qPCR results are presented as mean fold changes with respect to vehicle-treated mice (control). Protein expression was normalized to GAPDH, the loading control. Data represents the means of n = 4 samples for each group. * *p* < 0.05, ** *p* < 0.01, *** *p* < 0.001 or **** *p* < 0.0001 as determined by unpaired Student’s *t*-test. Ctl: control; R10: Rotenone 10 mg/kg; D1: dopamine-1-receptor; D2: dopamine-2-receptor; D3: dopamine-3-receptor; D4: dopamine-4-receptor; D5: dopamine-5-receptor; BDNF: brain-derived neurotrophic factor; GAPDH: glyceraldehyde 3-phosphate dehydrogenase; kDa: Kilodalton.

Interestingly, compared with extra-nigral regions, nigrostriatal regions reported little disruptions to dopamine receptors expression despite using the highest rotenone dosage (10 mg/kg) (Figure 3). 

For instance, the D1 receptor was only significantly altered in the spinal cord following rotenone treatment (10 mg/kg) compared to controls (** *p* = 0.0075; F (7, 7) = 2.160; Figure 3jj). Similarly, the D2 receptor was significantly reduced in the amygdala following rotenone treatment (* *p* = 0.0437; F (5, 7) = 5.629; Figure 3w). Rotenone treatment also decreased the expression of D3 receptor transcripts in the amygdala (** *p* = 0.0047; F (7, 7) = 6.073; Figure 3x), hippocampus (* *p* = 0.0240; F (7, 7) = 7.062; Figure 3ee), and spinal cord (* *p* = 0.0245; F (7, 7) = 1.651; Figure 3ll), with no changes observed in the midbrain (Figure 3c), striatum (Figure 3j) or prefrontal cortex (Figure 3q). The expression of D4 transcripts was decreased in the midbrain (*** *p* = 0.0006; F (7, 7) = 40.99; Figure 3d), amygdala (** *p* = 0.0014; F (7, 7) = 5.461; Figure 3y), hippocampus (*** *p* = 0.0001; F (7, 7) = 1.641; Figure 3ff) and spinal cord (** *p* = 0.0033; F (7, 7) = 5.910; Figure 3mm). The opposite was reported in the prefrontal cortex, with an upregulation of D4 mRNA in rotenone-treated mice (** *p* = 0.0041; F (7, 7) = 5.601; Figure 3r). Lastly, the D5 receptor transcripts were significantly decreased by rotenone treatment in the amygdala (** *p* = 0.0057; F (7, 7) = 5.488; Figure 3z). 

Several studies have linked dopamine, and dopamine receptors to brain-derived neurotrophic factor (BDNF) [35,36]. Our findings demonstrate that BDNF transcripts were up-regulated in response to both 3 and 10 mg/kg of rotenone in the midbrain (** *p* = 0.0012 and **** *p* < 0.0001, 3 mg/kg and 10 mg/kg rotenone, respectively, F (3, 26) = 18.84; Figure 3f) and prefrontal cortex (* *p* = 0.0338 and *** *p* = 0.0004, 3 mg/kg and 10 mg/kg rotenone, respectively, F (3, 28) = 7.609; Figure 3t). Interestingly, at the protein level, BDNF was significantly reduced in the midbrain following rotenone 10 mg/kg (*** *p* = 0.0005; F (3, 3) = 1.904; Figure 3g), while no changes in protein were observed in the prefrontal cortex (*p* > 0.05; F (3, 3) = 2.102; Figure 3u). In the striatum, the only significant increase in BDNF transcripts was seen in mice treated with 1 mg/kg rotenone (** *p* = 0.0075, F (3, 20) = 4.730; Figure 3m). Despite none of the rotenone dosages were able to affect BDNF transcripts in the amygdala (*p* > 0.05, F (3, 19) = 1.471; Figure 3aa), 10 mg/kg rotenone caused a significant downregulation of BDNF protein (**** *p* < 0.0001; F (3, 3) = 2.991; Figure 3bb). In contrast, all the dosages of rotenone caused a significant down-regulation of BDNF transcripts in the hippocampus (*** *p* = 0.0006, *** *p* = 0.0004 and ** *p* = 0.0012, at 1, 3 and 10 mg/kg rotenone, respectively, F (3, 27) = 9.228; Figure 3hh), however this did not translate to a reduction in BDNF protein (*p* > 0.05; F (3, 3) = 14.82; Figure 3ii). Lastly, we report a significant reduction of BDNF transcripts in the spinal cord of mice treated with 1 mg/kg (** *p* = 0.0073) and 3 mg/kg (** *p* = 0.0037) (F (3, 12) = 14.26; Figure 3oo), This correlated with a significant reduction of BDNF protein in the spinal cord of mice treated with 10 mg/kg rotenone (** *p* = 0.0052; F (3, 3) = 3.108; Figure 3pp). 

### 3.4. Rotenone Triggers CNS Region-Specific Changes in the Expression of Mitochondrial/Oxidative Stress Markers

**Figure 4:**
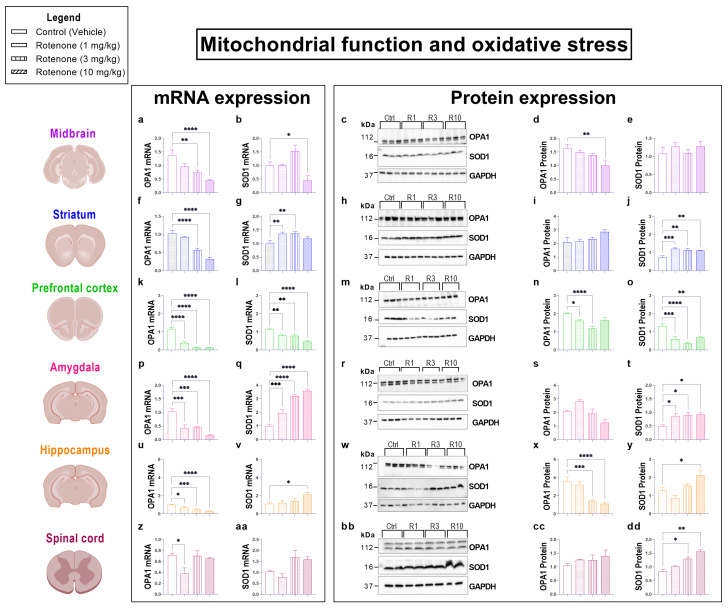
Rotenone increases oxidative stress in the brain and spinal cord. Oxidative stress was assessed by measuring the mRNA and protein expression of the mitochondrial marker OPA1 and the anti-oxidant enzyme SOD1. Real-time qPCR analyses of OPA1 and SOD1 mRNA expression in the midbrain (**a**,**b**), striatum (**f**,**g**), prefrontal cortex (**k**,**l**), amygdala (**p**,**q**), hippocampus (**u**,**v**) and spinal cord (**z**,**aa**) of mice administered with increasing dosages of rotenone (1, 3 and 10 mg/kg BW, i.p. per 21 days). Fold-changes were calculated using the ΔΔCt method after normalization to s18 (ribosomal protein s18 gene), the housekeeping gene. Each data point represents the mean value from n = 4 mice per each group. Representative Western blots and densitometry of OPA1 and SOD1 protein expression in the midbrain (**c**–**e**), striatum (**h**–**j**), prefrontal cortex (**m**–**o**), amygdala (**r**–**t**), hippocampus (**w**–**y**) and spinal cord (**bb**–**dd**) of mice exposed to the same treatment regimen as for mRNA studies. Protein expression was normalized to GAPDH, the loading control. Densitometric results are expressed as mean ± S.E.M from n = 4 mice per each group. * *p* < 0.05, ** *p* < 0.01, *** *p* < 0.001 or **** *p* < 0.0001 as determined by one-way ANOVA followed by Dunnett’s post hoc test. Ctrl: control; R1, 3, 10: Rotenone (1, 3, 10 mg/kg); OPA1: Mitochondrial dynamin like GTPase; SOD1: superoxide dismutase 1; GAPDH: glyceraldehyde 3-phosphate dehydrogenase; kDa: Kilodalton.

Rotenone is a mitochondrial complex I inhibitor [8] and prolonged treatment is thought to increase the levels of oxidative stress in the SNpc [37,38]. Here, we set out to investigate if rotenone altered the expression of the mitochondrial protein, OPA1 and the antioxidant enzyme SOD1 (Figure 4) in the midbrain, striatum and extra-nigral regions of the CNS (i.e., prefrontal cortex, amygdala, hippocampus and spinal cord) in C57BL/6 mice after administration with increasing doses of rotenone (1, 3 and 10 mg/kg). 

Analyses of OPA1 transcripts revealed that both 3 mg/kg and 10 mg/kg rotenone (but not the 1 mg/kg) significantly down-regulated OPA1 mRNA expression in the midbrain (** *p* = 0.0033 vs. vehicle [3 mg/kg rotenone], and **** *p* < 0.0001 [10 mg/kg rotenone]; F (3, 28) = 9.736; Figure 4a) and striatum (**** *p* < 0.0001, 3 mg/kg and 10 mg/kg rotenone, respectively; F (3, 28) = 33.67; Figure 4f). All rotenone dosages down-regulated OPA1 mRNA expression in the prefrontal cortex (**** *p* < 0.0001; F (3, 28) = 44.48; Figure 4k), amygdala (*** *p* = 0.0002 [1 mg/kg]; *** *p* = 0.0004 [3 mg/kg] and **** *p* < 0.0001 [10 mg/kg rotenone]; F (3, 20) = 18.66; Figure 4p) and hippocampus (* *p* = 0.0378 [1 mg/kg]; *** *p* = 0.0007 [3 mg/kg] and **** *p* < 0.0001 [10 mg/kg rotenone]; F (3, 27) = 12.17; Figure 4u). In the spinal cord, moderate but significant reductions of OPA mRNA expression were observed after exposure to 1 mg/kg of rotenone (* *p* = 0.0113 vs. vehicle; F (3, 20) = 4.603; Figure 4z), but not with higher dosages. 

Semi-quantitative analyses of OPA1 protein expression showed distinct patterns of reduction across the interrogated CNS regions. Notably, we report significantly reduced OPA1 expression in the midbrain only at 10 mg/kg rotenone (** *p* = 0.0070; F (3, 20) = 4.351; Figure 3c,d), whereas similar effects were seen in the hippocampus at both 3 mg/kg (*** *p* = 0.0004) and 10 mg/kg (**** *p* < 0.0001; F (3, 20) = 14.40; Figure 4w,x). In the prefrontal cortex, OPA1 protein expression was reduced in mice administered with 1 mg/kg (* *p* = 0.0263) and 3 mg/kg rotenone (**** *p* < 0.0001, F (3, 20) = 13.08; Figure 4m,n). 

Real-time qPCR analyses of SOD1 mRNA levels in the midbrain revealed significant reductions only in mice that received 10 mg/kg rotenone (* *p* = 0.0472; F (3, 28) = 8.201; Figure 4b). In contrast, lower dosages of rotenone were sufficient to induce a significant up-regulation of SOD1 transcripts in the striatum (** *p* = 0.0087 and ** *p* = 0.0049, 1 and 3 mg/kg rotenone, respectively; F (3, 28) = 5.224; Figure 4g) and amygdala (** *p* = 0.0013 and **** *p* < 0.0001, 1 and 3 mg/kg rotenone, respectively; F (3, 28) = 32.29; Figure 4q). The prefrontal cortex and amygdala were the two only regions in which both 1 mg/kg (** *p* = 0.0064 and *** *p* = 0.0002), 3 mg/kg (** *p* = 0.005 and *** *p* = 0.0004) and 10 mg/kg rotenone significantly increased SOD1 mRNA expression (**** *p* < 0.0001 for both regions, F (3, 28) = 14.98 [prefrontal cortex] and F (3, 20) = 18.66 [amygdala]; Figure 4l,q). Lastly, in the hippocampus, only the highest dose of rotenone (10 mg/kg) reliably up-regulated SOD1 mRNAs (* *p* = 0.0253; F (3, 28) = 3.133; Figure 4v). 

Notably, at the protein level, midbrain SOD1 expression was unaffected by any rotenone dosage (*p* = 0.5788; F (3, 32) = 0.6665; Figure 4c,e). In contrast, SOD1 protein levels were remarkably altered by rotenone in the other CNS regions tested. Specifically, rotenone significantly increased the expression of SOD1 at all dosages both in the striatum (*** *p* = 0.0002, ** *p* = 0.0011 and ** *p* = 0.0022, 1, 3 and 10 mg/kg, respectively. F (3, 20) = 10.11; Figure 4h,j) and amygdala (* *p* = 0.0270, * *p* = 0.0161 and * *p* = 0.0137, 1, 3 and 10 mg/kg, respectively; F (3, 8) = 6.486; Figure 4r,t). A dose-dependent increase in SOD1 protein expression was observed in the hippocampus, although levels were significantly up-regulated at 10 mg/kg (* *p* = 0.0136, 10 mg/kg rotenone; F (3, 20) = 7.811; Figure 4w,y) and in the spinal cord (* *p* = 0.0156 and ** *p* = 0.0030 3 mg/kg and 10 mg/kg rotenone, respectively; F (3, 4) = 25.08; Figure 4bb,dd).

### 3.5. Rotenone-Induced Neuroinflammation Is Confined to the Midbrain and Inhibited in Extra-Nigral CNS Regions

**Figure 5:**
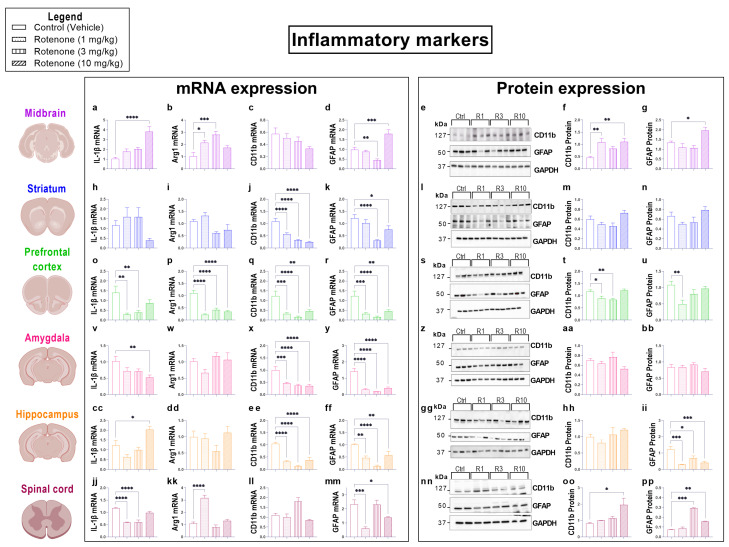
Rotenone-induced neuroinflammation is restricted to the midbrain and inhibited in extra-nigral CNS regions. Real-time qPCR analyses of IL-1β, Arg1, CD11b, GFAP mRNA expression in the midbrain (**a**–**d**), striatum (**h**–**k**), prefrontal cortex (**o**–**r**), amygdala (**v**–**y**), hippocampus (**cc**–**ff**) and spinal cord (**jj**–**mm**) of mice treated with either 1, 3 and 10 mg/kg rotenone i.p. for 21 days. Fold changes were calculated using the ΔΔCt method after normalization to s18 ribosomal protein subunit gene. Data represents means of n = 4 samples for each group. Western blot and densitometric analysis of CD11b and GFAP protein expression in the midbrain (**e**–**g**), striatum (**l**–**n**), prefrontal cortex (**s**–**u**), amygdala (**z**–**bb**), hippocampus (**gg**–**ii**) and spinal cord (**nn**–**pp**). Densitometric results are expressed as mean ± S.E.M of 4–6 samples per group. * *p* < 0.05, ** *p* < 0.01, *** *p* < 0.001 or **** *p* < 0.0001 as determined by one-way ANOVA followed by Dunnett’s post hoc test. Ctrl: control; R1, 3, 10: Rotenone (1, 3, 10 mg/kg); GFAP: Glial Fibrillary Acidic Protein; IL-1β: Interleukin-1-beta; Arg1: Arginase 1; GAPDH: Glyceraldehyde 3-phosphate Dehydrogenase; kDa: Kilodalton.

In order to define whether chronic rotenone treatment caused diffuse neuroinflammation, we interrogated the gene expression of inflammatory mediators as well as the gene and protein expression of glial activation markers in the same CNS regions indicated above. To our surprise, we report that rotenone-induced neuroinflammation is restricted to the midbrain. In contrast, a global down-regulation of the expression of the pro-inflammatory cytokine IL-1β and of two distinct glial activation markers was seen in extra-nigral CNS regions. (Figure 5). 

Real-time qPCR analyses of the pro-inflammatory cytokine IL-1β demonstrated that gene expression was significantly up-regulated in the midbrain (**** *p* < 0.0001, F (3, 19) = 16.79; Figure 5a) and, to a lesser extent, in the hippocampus of mice treated with the highest dose of rotenone (10 mg/kg) (* *p* = 0.0119, F (3, 27) = 10.40; Figure 5cc). Conversely, a significant reduction of IL-1β gene expression was observed both in the prefrontal cortex (** *p* = 0.0014 and ** *p* = 0.0036 at 1 mg/kg and 3 mg/kg, respectively, F (2, 28) = 6.537; Figure 5o) and spinal cord at the lower dosages (**** *p* < 0.0001 at both 1 and 3 mg/kg, respectively, F (3, 19) = 26.68; Figure 5jj), but not in animals treated with 10 mg/kg rotenone (*p* > 0.05, Figure 5o,jj).

Gene expression of the anti-inflammatory marker Arg1 was altered in three distinct CNS regions in response to rotenone. Notably, in the midbrain, both 1 mg/kg and 3 mg/kg rotenone significantly up-regulated Arg1 transcripts (* *p* = 0.0149 and *** *p* = 0.0002, respectively, F (3, 22) = 8.791; Figure 5b). Unexpectedly, Arg1 transcripts were unchanged in mice treated with 10 mg/kg rotenone. In the prefrontal cortex, Arg1 was markedly down-regulated at all the dosages tested (**** *p* < 0.0001, F (3, 20), **** *p* <0.0001; Figure 5p). Lastly, rotenone up-regulated the expression of Arg1 in the spinal cord at the lowest dosage tested (1 mg/kg) (**** *p* < 0.0001, F (3, 12) = 38.78; Figure 5kk). 

Expression of CD11b, a macrophage/microglial activation marker, was measured both at the mRNA and protein level. In the midbrain, CD11b transcripts were not affected by any of the rotenone dosages tested (*p* = 0.1397, F (3, 16) = 2.106; Figure 5c). In contrast, CD11b protein expression was significantly increased in mice that received 1 mg/kg and 10 mg/kg rotenone (** *p* = 0.0016 and ** *p* = 0.0011, respectively, F (3, 32) = 6.879; Figure 5e,f). Similarly, to the midbrain, we report no changes in CD11b transcripts in the spinal cord (*p* > 0.05, F (3, 20) = 8.855; Figure 5ll); however, these were paralleled by a significant increase in CD11b protein levels, but only at the highest dose of rotenone (* *p* = 0.0447, F (3, 4) = 5.433; Figure 5nn,oo). In the prefrontal cortex, both CD11b mRNA and protein expression was consistently down-regulated. Specifically, CD11b transcripts were significantly reduced at all the dosages tested (*** *p* = 0.0003, **** *p* < 0.0001 and ** *p* = 0.0017, 1, 3, and 10 mg/kg rotenone, respectively; F (3, 28) = 11.21; Figure 5q), and CD11b protein levels were reduced at both 1 and 3 mg/kg rotenone (* *p* = 0.0329 and ** *p* = 0.0099, F (3, 20) = 7.376; Figure 5s,t). Finally, CD11b mRNA levels were robustly reduced at every dose tested both in the striatum (**** *p* < 0.0001, F (3, 28) = 31.31; Figure 5j) amygdala (*** *p* = 0.0003 at 1 mg/kg; **** *p* < 0.0001 at 3 and 10 mg/kg, F (3, 28) = 13.38; Figure 5x) and hippocampus (**** *p* < 0.0001, F (3, 28) = 39.28; Figure 5ee). However, none of the mRNA results in these regions were corroborated by protein expression data (* *p* = 0.0115 [striatum], F (3, 32) = 4.316; Figure 5m; *p* = 0.0958 [amygdala], F (3, 8) = 2.990; Figure 5aa; *p* = 0.2207 [hippocampus], F (3, 20) = 1.601; Figure 5hh).

Analyses of GFAP expression, an astrocyte-specific marker, revealed a moderate but significant reduction of GFAP mRNAs in the midbrain of mice exposed 3 mg/kg rotenone (** *p* = 0.009, F (3, 26) = 19.12; Figure 5d), followed by a significant increase at the highest dosage of rotenone (*** *p* = 0.0004, Figure 5d). These results were corroborated by similar changes at the protein level (* *p* = 0.017, F (3, 32) = 7.865; Figure 5e,g). Parallel experiments in extra-nigral regions identified a generalized down-regulation of GFAP transcripts (Figure 5k,r,y,ff). In the striatum, GFAP transcripts were significantly down-regulated at 3 mg/kg and 10 mg/kg rotenone (**** *p* < 0.0001 and * *p* = 0.0443, respectively, F (3, 28) = 9.433; Figure 5k), whereas GFAP protein expression was reliably reduced at all the dosages tested (** *p* = 0.0069, * *p* = 0.02 and * *p* = 0.037 at 1, 3, and 10 mg/kg rotenone, respectively, F (3, 20) = 2.652; Figure 5l,n). In the prefrontal cortex, GFAP mRNAs were robustly reduced by all the rotenone dosages (*** *p* = 0.0003, **** *p* < 0.0001 and ** *p* = 0.0017 at 1, 3, and 10 mg/kg rotenone, respectively, F (3, 28) = 11.21; Figure 5r). However, GFAP protein expression was significantly reduced only with 1 mg/kg rotenone (** *p* = 0.0039), whereas it was marginally reduced at higher concentrations (*p* > 0.05, F (3, 20) = 5.156; Figure 5s,u). In the amygdala, GFAP mRNA expression was thoroughly reduced by rotenone administration at all dosages tested (**** *p* < 0.0001, F (3, 25) = 33.18; Figure 5y); however, these results were not supported by protein expression data (*p* = 0.3877, F (3, 20) = 1.061; Figure 5z,bb). In contrast, hippocampal GFAP transcripts were significantly reduced following rotenone treatment (** *p* = 0.001, **** *p* < 0.0001 and ** *p* = 0.0093 at 1, 3, and 10 mg/kg rotenone, respectively, F (3, 28) = 14.15; Figure 5ff). Similarly, hippocampal GFAP protein expression was reduced at different levels by rotenone (*** *p* = 0.0001, * *p* = 0.0157 and *** *p* = 0.0005 at 1, 3, and 10 mg/kg rotenone, respectively, F (3, 20) = 10.72; Figure 5gg,ii). In the spinal cord, GFAP mRNA expression was significantly down-regulated by all rotenone treatments (* *p* < 0.05 for all dosages, F (3, 12) = 12.22; Figure 5mm); however, these changes were not seen at the protein level (*p* > 0.05, F (3, 4) = 140.2; Figure 5nn,pp).

### 3.6. Rotenone Intoxication Causes a Global Disruption in the Expression of Neuropeptides in the CNS

In view of the protective role elicited by endogenous neuropeptides in preventing neuronal deterioration in PD and other neurodegenerative disorders [39,40,41], we sought to determine if rotenone intoxication interfered with the expression of well-established neuropeptides/neurotrophic molecules. As such, we measured transcript levels of ADNP, PACAP and VIP in the CNS of mice that received increasing dosages of rotenone. In addition, for both PACAP and VIP neuropeptides, we also measured protein expression.

**Figure 6:**
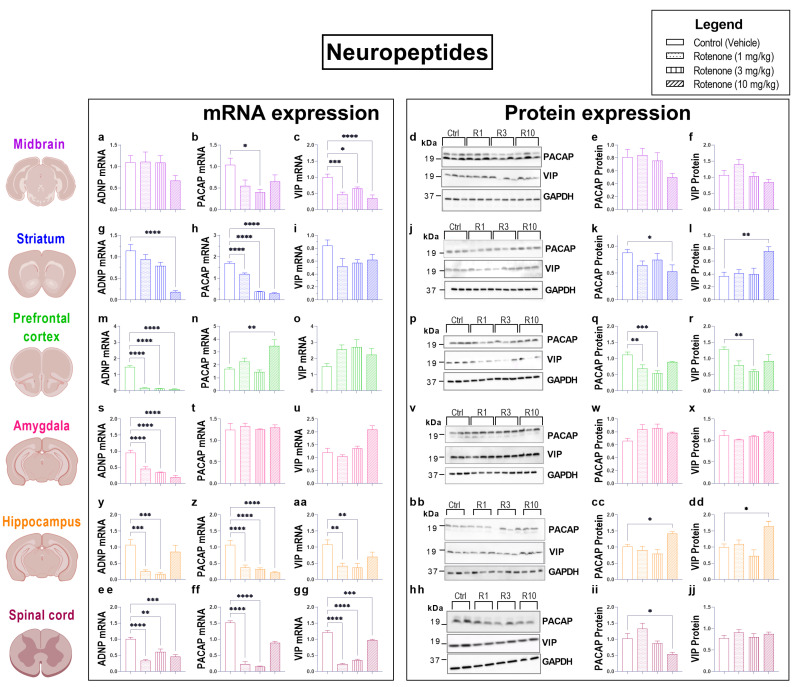
Rotenone disrupts the expression of neuropeptides in the CNS. Real-time qPCR analyses of ADNP, PACAP and VIP mRNA expression in the midbrain (**a**–**c**), striatum (**g**–**i**), prefrontal cortex (**m**–**o**), amygdala (**s**–**u**), hippocampus (**y**–**aa**) and spinal cord (**ee**–**gg**) of mice treated with either 1, 3 and 10 mg/kg rotenone i.p. for 21 days. Fold changes were calculated using the ΔΔCt method after normalization to s18 ribosomal protein subunit gene. Data represents means of n = 4 samples for each group. Western blot and densitometric analyses of PACAP and VIP protein expression in the midbrain (**d**–**f**), striatum (**j**–**l**), prefrontal cortex (**p**–**r**), amygdala (**v**–**x**), hippocampus (**bb**–**dd**) and spinal cord (**hh**–**jj**). Densitometric results are expressed as mean ± S.E.M of 4–6 samples per group. * *p* < 0.05, ** *p* < 0.01, *** *p* < 0.001 or **** *p* < 0.0001 as determined by one-way ANOVA followed by Dunnett’s post hoc test. Ctrl: control; R1, 3, 10: Rotenone (1, 3, 10 mg/kg); PACAP: pituitary adenylate cyclase-activating peptide; VIP: vasoactive intestinal peptide; ADNP: activity-dependent neurotrophic peptide; GAPDH: glyceraldehyde 3-phosphate dehydrogenase; kDa: Kilodalton.

Analyses of ADNP mRNA expression in the CNS revealed a global decrease in transcript levels across the regions examined. With the exception of the midbrain, where we do not report significant changes in ADNP expression (*p* = 0.3259, F (3, 26) = 1.210; Figure 6a), all rotenone treatments significantly down-regulated ADNP transcripts both in the prefrontal cortex (**** *p* < 0.0001, F (3, 20) = 238.3; Figure 6m) and amygdala (**** *p* < 0.0001, F (3, 27) = 30.65; Figure 6s).

A similar down-regulation was observed in the spinal cord at 1 mg/kg (**** *p* < 0.0001), 3 mg/kg (** *p* = 0.003) and 10 mg/kg (*** *p* = 0.0003) (F (3, 12) = 19.10; Figure 6ee). In the striatum, ADNP transcripts dose-dependently decreased; however, they were statistically significant only in mice that were treated with 10 mg/kg of rotenone (**** *p* < 0.0001, F (2, 28) = 16.67; Figure 6g). Conversely, only the two lowest doses of rotenone caused a significant decrease in ADNP mRNA expression in the hippocampus (*** *p* = 0.0004 and *** *p* = 0.0001 at 1 mg/kg and 3 mg/kg rotenone, respectively, F (3, 28) = 11.52; Figure 6w). 

When looking at PACAP transcripts, we found significantly down-regulated mRNA levels in the midbrain (Figure 6b), striatum (Figure 6h), hippocampus (Figure 6z) and spinal cord (Figure 6ff), but not in the prefrontal cortex or amygdala (*p* = 0.6542, F (3, 20) = 0.392; Figure 6n) (*p* = 0.8957, F (3, 20) = 0.1992; Figure 6t). In the midbrain, all dosages of rotenone reduced PACAP mRNA levels, although a significant reduction was seen only with 3 mg/kg rotenone (* *p* = 0.0143, F (3, 21) = 3.292; Figure 6b). Consistently, midbrain PACAP protein expression was significantly reduced both at 3 and 10 mg/kg (* *p* < 0.05 for both, F (3, 16) = 1.778; Figure 6d,e). In the striatum, PACAP transcripts were robustly reduced by all rotenone dosages (**** *p* < 0.0001, F (3, 20) = 264.4; Figure 6h); however, PACAP protein levels were only slightly reduced until the highest rotenone dosage (* *p* = 0.0495, F (3, 20) = 2.322; Figure 6k,l). Interestingly, whereas PACAP mRNAs were not affected by rotenone treatment in the prefrontal cortex (Figure 6n), protein expression was significantly reduced both at 1 mg/kg (** *p* = 0.0039) and 3 mg/kg (*** *p* = 0.0002) and marginally at 10 mg/kg (*p* > 0.05, F (3, 20) = 9.409; Figure 6q,r). In the amygdala, consistent with PACAP mRNA measurements (Figure 6t), there were no significant changes in PACAP protein expression with any rotenone dosages (*p* > 0.05, F (3, 16) = 3.778; Figure 6w,x), nor there were changes in hippocampal PACAP protein levels (*p* > 0.05, F (3, 20) = 7.238; Figure 6cc,dd), except at the highest dosages (* *p* < 0.05). In the spinal cord, only 1 mg/kg and 3 mg/kg (**** *p* < 0.0001; F (3, 12) = 138.8; Figure 6ff) caused a significant reduction in PACAP transcripts, while only 10 mg/kg rotenone significantly downregulated PACAP protein expression (* *p* = 0.0674; F (3, 12) = 7.226; Figure 6hh,ii).

VIP transcript levels were significantly diminished in the midbrain, hippocampus and spinal cord (Figure 6aa,gg), but not in the striatum (*p* = 0.0841, F (3, 20) = 2.556; Figure 6i), prefrontal cortex (*p* = 0.0896, F (3, 20) = 2.491; Figure 6o) or amygdala (*p* = 0.0956, F (3, 20) = 14.73; Figure 6u). Specifically, midbrain VIP mRNAs were down-regulated in response to the different rotenone dosages (*** *p* = 0.0002, * *p* = 0.0183, **** *p* < 0.0001 at 1, 3 and 10 mg/kg rotenone, respectively, F (3, 22) = 12.38; Figure 6c). In contrast, VIP protein expression was not affected by rotenone in this brain region (*p* = 0.0692 [midbrain], F (3, 18) = 2.805; Figure 6d,f), neither it was in the striatum, amygdala or spinal cord (*p* > 0.05 [striatum], F (3, 20) = 6.219; Figure 6l; *p* = 0.2811 [amygdala], (F (3, 12) = 1.435; Figure 6x; *p* > 0.05 [spinal cord], F (3, 20) = 16.35; Figure 6jj). Similarly, to PACAP, VIP expression was only reduced at the protein level in the prefrontal cortex, with significantly reduced expression at the two highest rotenone dosages tested (** *p* = 0.0062 and * *p* < 0.05 at 3 and 10 mg/kg, respectively, F (3, 20) = 4.355; Figure 6p,r), whilst it was increased in the hippocampus, but only at the highest dosage (* *p* < 0.05 at 10 mg/kg, F(3, 20) = 7.076). Hippocampal VIP mRNA expression was reduced by rotenone, with significant reductions at 1 and 3 mg/kg (** *p* = 0.0032 and ** *p* = 0.0015, respectively, F (3, 28) = 6.428; Figure 6aa). Similarly, only VIP transcripts were reduced in the spinal cord by all rotenone dosages (**** *p* < 0.0001 at 1 and 3 mg/kg, and *** *p* = 0.0008 at 10 mg/kg rotenone, F (3, 20) = 158.0; Figure 6gg), whereas protein levels remained unchanged (*p* > 0.05 at all dosages, F (3, 12) = 0.7894; Figure 6hh–jj).

## 4. Discussion

In this study, we sought to provide a comprehensive analysis of the behavioural and neurochemical alterations caused by rotenone intoxication in C57BL/6 mice, which are summarized in Table 3. For this purpose, animals were injected daily with rotenone at increasing dosages for a period of 21 days via intraperitoneal route. We opted to utilize this route of administration as this has been found to be the most effective to achieve a PD-like phenotype, at least in mice. Whereas a large bulk of studies have demonstrated that rotenone intoxication reliably mimics human PD pathology [12,13,14], several discrepancies have been identified when comparing studies using different administration routes. For instance, a study reported that dopamine loss and behavioural deficits were seen in mice that were administered with 5 mg/kg rotenone by oral gavage for 12 weeks [42]. Another study using the same route of administration reported that dosages as high as 30 mg/kg rotenone for periods of 28 or 56 days were required to obtain similar nigrostriatal degeneration and locomotor impairment [10]. In contrast, studies in which rotenone was administered via intraperitoneal route have shown that lower dosages of rotenone (0.75–3 mg/kg) and relatively shorter exposure periods (up to 3 weeks) were sufficient to achieve similar outcomes [15,43]. These results suggest that, at least in mice, rotenone adsorption via the gastrointestinal tract might not be as efficient as in rats. 

A large portion of our investigations was to assess if rotenone toxicity disrupted the expression of dopamine, oxidative stress and inflammatory markers, as well as neuropeptides and trophic factors in the midbrain and in extra-nigral CNS regions. The reason behind these investigations was based on the ever-growing repertoire of behavioural alterations that are seen in patients with PD, which are not limited to the locomotor impairment associated with nigro-striatal degeneration. Our data indicates that rotenone toxicity causes a broad spectrum of neurochemical alterations, such as changes in the expression of dopamine, oxidative stress and inflammatory markers, as well as neurotrophic factors/neuropeptides, some of which extend beyond the canonical nigrostriatal pathway. These results provide evidence of distinct and CNS region-specific neurochemical alterations at multiple CNS levels in rotenone intoxicated mice.

### 4.1. Toxic Effects of Rotenone in Extra-Nigral Regions—General Considerations

Several CNS regions are altered in PD pathogenesis; however, most PD studies focus on the nigral-striatal pathway and pay little attention to extra-nigral changes. This is surprising as non-motor symptoms of PD are known to clinically manifest much earlier than the classical motor symptoms associated with degeneration of dopaminergic neurons in the SNpc. For example, neuroimaging studies revealed structural and functional changes in the limbic cortico-striato-thalamocortical circuits that correlated with the high prevalence of anxiety in PD [44]. Further imaging studies demonstrated atrophy in the gray matter, caudate, putamen, nucleus accumbens and amygdala and associated atrophy of these regions with the cognitive impairment observed in PD [45]. Villar-Conde and colleagues revealed synaptic changes in the hippocampus of PD patients [46], aligning with an increased dementia risk in PD patients. Apathy is one of the most common PD-related mood changes and has been linked to alterations in the medial and lateral prefrontal cortex and the limbic system [47]. Finally, the spinal cord, an often neglected part of the CNS in the context of PD, has been shown to exhibit alpha-synucleinopathy and could be the origin of several non-motor symptoms, including urinary, sexual, gastrointestinal dysfunctions and pain [48]. PD is a very heterogeneous disease that presents differently in each patient. Accordingly, PD research should not be restricted to the nigro-striatal pathway, as this study and others have contributed to reveal a unique profile of extra-nigral regions that could help inform early diagnosis and clinical management of disease.

### 4.2. Rotenone Impairs Locomotor and Exploratory Behaviours

First, we evaluated the effects of rotenone on body weight and locomotor and exploratory behaviour. Mouse rotenone models have previously shown that the toxicant does not induce significant weight loss in rats. As shown by Indel et al., they found that there was no difference in weight when comparing C57BL/6 mice that received 30 or 100 mg/kg rotenone for 56 days with controls [2]. These results align with our study, as rotenone did not cause any significant weight loss; however, mice gained less weight compared to saline-injected controls. Assessments of locomotor and exploratory behaviours unveiled a dose-dependent deterioration of both behavioural domains, although these were best appreciated by measures of locomotor behaviour. A similar outcome has been highlighted in several studies, where rotenone reliably reduced the total distance travelled and number of times mice moved between areas of the OF [38,49]. This finding is consistent with previous studies demonstrating that intraperitoneal injections of 3 mg/kg rotenone daily for 21 days induced locomotor impairment in C57BL/6 mice [43]. Furthermore, studies report that at least a 3-week intoxication protocol is required to attain behavioural deficits [37], in agreement with our study, where the most consistent induction of locomotor and exploratory impairments was seen on day 21. 

### 4.3. Rotenone Reduces the Expression of Dopaminergic Markers

Next, we confirmed if the behavioural impairments triggered by rotenone were associated with the deterioration of dopaminergic pathway. As expected, our study reproduced the selective decline of dopaminergic neurons within the midbrain and striatum seen in other studies [37,43]. However, rotenone dosages in our study were about ten times lower than that used in orally administered mice to achieve similar outcomes [37]. Investigations were also extended to the spinal cord, another CNS region that also seem to be vulnerable to the effects of rotenone intoxication [50]. In this region, TH and DAT transcripts and protein levels were reduced, especially with the highest rotenone dosage. This result is, to some extent, similar to that reported in mutant A53T mice (a genetic model of PD), where axonal degeneration and motor neuron cell loss also extended to the spinal cord of these mice [51]. This is particularly important, as some studies have already reported some degree of association between spinal cord damage and the autonomic dysfunctions seen in PD [52,53,54]. Interestingly, we report that extra-nigral regions such as the prefrontal cortex, amygdala, hippocampus and spinal cord were the only CNS regions that demonstrated significant changes in the expression of the dopamine receptors. These receptors were analysed due to their known alterations during PD pathogenesis [55]. Apparently, it can be inferred that damage to the dopaminergic system in PD may not be limited to the nigrostriatal pathway, although more in-depth investigations are needed to understand the involvement of extra-nigral circuits and spinal dopaminergic system damage in the development of motor and non-motor symptoms. Additionally, the expression of brain-derived neurotrophic factor (BDNF) was reduced in several CNS regions, including the midbrain, amygdala and spinal cord, suggesting neurotoxic damage in these areas. However, it should be highlighted that BDNF mRNA and protein levels (as well as other markers interrogated in this study) produced some discordant results. We believe this can be attributed to several factors including: post-transcriptional and post-translational modifications, short half-life of proteins in vivo, different turnover of mRNA vs. protein at time of tissue harvesting and other factors not mentioned here. 

### 4.4. Systemic Rotenone Triggers Widespread Signs of Oxidative Stress

Rotenone is a known inhibitor of mitochondrial complex I [13,56], which can trigger oxidative stress damage in the CNS. Here, we interrogated OPA1, a mitochondrial fusion protein and SOD1, an antioxidant enzyme, as two markers of mitochondrial function and oxidative stress in the CNS [57,58]. Our results revealed disturbances in both OPA1 and SOD1 expression in all regions tested, suggesting that rotenone toxicity causes redox dysfunctions that extended to various CNS regions. Systemic neurotoxicity of rotenone has been reported in numerous studies [10,50,59]. Furthermore, clinical imaging studies in PD patients have identified the coexistence of structural and functional alterations in extra-nigral regions such as the hippocampus [60], amygdala and prefrontal cortex [44]. The evidence of widespread increase in oxidative stress markers in regions outside of the nigrostriatal pathway in our PD model support the idea that such neurochemical changes might be the consequence (or cause) of structural changes. This could be clinically relevant, as there is some evidence suggesting that non-motor symptoms often precede motor symptoms in the prodromal stages of the disease [5]. 

### 4.5. Rotenone-Induced Inflammation Is Restricted to the Midbrain

Neuroinflammation has become a well-established player in PD pathogenesis [61,62]. Rotenone has previously been shown to induce inflammation, with many studies utilizing rotenone reporting an association between oxidative stress and neuroinflammation as co-conspirators in PD pathogenesis [63]. Zhang and colleagues provided evidence of microglial activation in hippocampal and cortical regions of mice and suggested that this contributed to the cognitive decline seen in their rotenone-induced mouse PD model [16]. Evidence of neuroinflammation in PD patients has been found in several brain regions, including the SNpc, striatum, hippocampus and cerebral cortex, as well as in bodily fluids such as the cerebrospinal fluid [63]. Surprisingly, in our study rotenone-induced inflammation was confined primarily to the midbrain. This is intriguing, especially given the findings reported above from other research groups. To make the scenario even more complex, we also found that the expression of most inflammatory and glial markers were globally down-regulated in all the other CNS structures tested. Whilst the latter could be considered as a direct effect of systemic toxicity, we cannot rule out that the stress response triggered by treatments (and behavioural tests) may have contributed to dampening the overall activity of the immune system, hence explaining why only a restricted pattern of inflammation was found in the midbrain. Other aspects to consider are the existence of conflicting data available in the literature on the ability of rotenone as an inducer of neuroinflammation. In fact, this seems to depend on several factors, including the route of administration and duration of treatment. In one study, oral administration of rotenone caused dopaminergic degeneration in the absence of marked changes in glial activation [37]. Conversely, rotenone infusion via an osmotic pump strongly activated both astrocytes and microglia in the SNpc and striatum [64]. A more recent study that included a 2-week period where neuroinflammation was allowed to progress after rotenone administration concluded that activation of glial cells appeared to drive neuronal loss following neurotoxic exposure to rotenone [65]. Altogether, these results suggest that variations to the experimental protocols used in rotenone intoxication models may produce signs of inflammation ranging from no inflammation to widespread inflammation in several CNS sites. 

### 4.6. Alterations of Neurotrophic Factors and Neuropeptides Expression Profiles in the Rotenone Mouse Model

Lastly, we analysed the expression of neuropeptides known to play critical roles in the modulation of neurological processes within the CNS. PACAP and VIP are two related neuropeptides that are expressed throughout the CNS that exert essential neuroprotective and immune modulatory roles [66]. PACAP knockout mice display age-related degenerative signs earlier than wild type animals, including increased neuronal vulnerability, systemic degeneration and increased inflammation, suggesting the importance of this peptide in maintaining a healthy and functional CNS [67]. VIP has been shown to prevent PD pathogenesis in several preclinical models of disease [68]. These peptides were analysed as we have previously shown that both peptides reduce microglial polarization in vitro [69], demonstrating their potential as therapeutic targets in PD. In a study by de Souza and collaborators, the authors describe these two peptides as neuroprotective and anti-inflammatory against experimental PD, acting mainly by reducing neuroinflammation, promoting dopaminergic neuronal survival and preserving cognitive functions [70]. Moreover, other evidence indicates that PACAP and BDNF [71] share similar neuroprotective pathways, as do VIP and ADNP [72]. Our results demonstrate these pathways are globally down-regulated in our rotenone model, suggesting that one of the modalities through which rotenone imparts damage to the CNS is via reducing the endogenous neuroprotective potential of these peptides. This correlates with studies that provide evidence on the therapeutic potential of PACAP, VIP, BDNF and ADNP in PD [39,41,73]. However, more research is needed to elucidate the exact role of these factors in PD pathogenesis. 

Like most models of neurodegenerative diseases, it is difficult to model PD, as we still do not have a defined clinical diagnostic criteria that enables early identification, with confirmation of clinical PD occurring from postmortem analyses of brain tissue. However, comparisons between genetic, neurotoxic and inflammatory models indicate that regardless of the initial trigger, the CNS is subjected to a cascade of pathological events that include inflammation, mitochondrial dysfunction, oxidative stress and dopaminergic neuronal loss [62]. However, the neurochemical changes reported here correlate with other PD models as well as clinical studies [48,60,74,75]. Altogether, our results suggest that rotenone induces multiple neurochemical alterations across different CNS sites, suggesting that this disease model may be useful to study certain PD domains, thereby providing an excellent scaffold to study the efficacy of novel compounds to target non-motor and motor symptoms of PD.

## Figures and Tables

**Table 1:** List of primer sets used in real-time qPCR analyses.

Accession #	Gene	Primer Sequence (5′–3′)	Length (bp)
NM_009377.2	Tyrosine hydroxylase (TH)	Fwd GCCCTACCAAGATCAAACCTAC Rev ATACGAGAGGCATAGTTCCTGA	93
NM_010020.3	Dopamine transporter (DAT)	Fwd ATGACATCAAGCAGATGACTGG Rev CACGACCACATACAGAAGGAAG	95
NM_010076.1	D1 receptor (D1)	Fwd GAGCAGGACATACGCCATTT Rev GCTTCTGGGCAATCCTGTAG	101
NM_010077.2	D2 receptor (D2)	Fwd GTCAACACCAAGCGTAGCAG Rev CGGTGCAGAGTTTCATGTCC	97
NM_007877	D3 receptor (D3)	Fwd GGGGTGACTGTCCTGGTCTA Rev AAGCCAGGTCTGATGCTGAT	110
NM_007878.2	D4 receptor (D4)	Fwd CTGCAGACACCCACCAACTA Rev CCTGGACCTCGGAGTAGACA	100
NM_013503.3	D5 receptor (D5)	Fwd TTGGGAGCTAGACGGGAGAA Rev CTGTGCAATGCGGTAGATGC	139
NM_001131020.1	Glial fibrillary acidic protein (GFAP)	Fwd GAGATTCGCACTCAATACGAGG Rev CTGCAAACTTAGACCGATACCA	79
NM_001082960.1	CD11b	Fwd GAGCAGGGGTCATTCGCTAC Rev GCTGGCTTAGATGCGATGGT	94
NM_008361.4	Interleukin-1β (IL-1β)	Fwd GCTACCTGTGTCTTTCCCGT Rev CATCTCGGAGCCTGTAGTGC	164
NM_007482.3	Arginase-1 (Arg1)	Fwd ACAAGACAGGGCTCCTTTCAG Rev TTAAAGCCACTGCCGTGTTC	105
NM_011434.2	Superoxide dismutase (SOD1)	Fwd CAATGGTGGTCCATGAGAAACA Rev CCCAGCATTTCCAGTCTTTGTA	77
NM_001199177.1	Mitochondrial dynamin like GTPase (OPA1)	Fwd GCCCTTCTCTTGTTAGGTTCAC Rev ACACCTTCCTGTAATGCTTGTC	88
NM_007540.4	Brain-derived neurotrophic factor (BDNF)	Fwd CGAGTGGGTCACAGCGGCAG Rev GCCCCTGCAGCCTTCCTTGG	160
NM_001310086.1	Activity-dependent neuroprotective protein (ADNP)	Fwd GTGACATTGGGTTGGAATACTGT Rev AGGTTTTGTCCGATAGTCCTGA	149
NM_016989.2	Pituitary adenylate-cyclase-activating polypeptide (PACAP)	Fwd AGGCTTACGATCAGGACGGA Rev CTCCTGTCGGCTGGGTAGTA	121
NM_053991.1	Vasoactive intestinal peptide (VIP)	Fwd CCTGGCGATCCTGACACTCT Rev CTGCAGCCTGTCATCCAACC	100
NM_213557.1	18S ribosomal subunit (s18)	Fwd GGCGGAAAATAGCCTTCGCT Rev AGCCCTCTTGGTGAGGTCAA	101

Forward and reverse primers were selected from the 5′ and 3′ region of each gene mRNA. The expected length of each amplicon is indicated in the right column. #: number.

**Table 2:** List of primary and secondary antibodies used in Western blot.

Antibody	Dilution	Source (Cat. #)
Tyrosine hydroxylase (TH)	1:500	Abcam (ab112)
Dopamine transporter (DAT)	1:1000	Abcam (ab128848)
Brain-derived neurotrophic factor (BDNF)	1:1000	GeneTex (GTX132621)
Glial fibrillary acidic protein (GFAP)	1:1000	Abcam (ab68428)
CD11b	1:1000	Abcam (ab133357)
Mitochondrial dynamin like GTPase (OPA1)	1:1000	GeneTex (GTX129917)
Superoxide dismutase (SOD1)	1:1000	GeneTex (GTX100554)
Pituitary adenylate-cyclase-activating polypeptide (PACAP)	1:1000	GeneTex (GTX37576)
Vasoactive intestinal peptide (VIP)	1:1000	GeneTex (GTX129461)
Glyceraldehyde-3-phosphate dehydrogenase (GAPDH)	1:1000	BioRad (VPA00187)
Goat anti-Rabbit IgG HRP	1:10,000	BioRad (STAR208P)
Goat anti-Mouse IgG (H+L)-HRP	1:10,000	BioRad (1706516)

#: number.

**Table 3:** Summary of results.

	Midbrain	Striatum	Prefrontal Cortex	Amygdala	Hippocampus	Spinal Cord
Dopaminergic markers						
TH	↓	n/a	n/a	n/a	n/a	↓
	↓	n/a	n/a	n/a	n/a	↓
DAT	n/a	↓	n/a	n/a	n/a	↓
	n/a	↓	n/a	n/a	n/a	↓
Dopamine receptors						
D1	-	-	-	-	-	-
D2	-	-	-	-	-	-
D3	-	-	-	↓	↓	↓
D4	↓	-	↑	↓	↓	↓
D5	-	-	-	↓	-	-
Brain-derived neurotrophic factor						
BDNF	↑	↑	↑	-	↓	↓
	↓	↑	-	↓	-	↓
Inflammation						
GFAP	↑	↓	↓	↓	↓	↓
	↑	-	↓	-	↓	↑
Iba1/CD11b	-	↓	↓	↓	↓	-
	↑	-	↓	-	-	↑
IL-1β	↑	-	↓	↓	↑	↓
Arg1	↑	-	↓	-	-	↑
Oxidative stress						
OPA1	↓	↓	↓	↓	↓	-
	↓	-	↓	-		-
SOD1	↓	↑	↑	↑	↑	-
	-	↑	↑	↑	↑	↑
Neuropeptides						
ADNP	-	↓	↓	↓	↓	↓
PACAP	↓	↓	↑	-	↓	↓
	-	↓	↓	-	↑	↓
VIP	↓	-	-	-	↓	↓
	-	↑	↓	-	↑	-

Arrows indicate the direction (↑ upregulation or ↓ downregulation; - indicates no change) of the observed changes in mRNA (top column) and protein expression (bottom column) across the CNS regions analysed. TH: tyrosine hydroxylase; DAT: dopamine transporter; D1: dopamine-1-receptor; D2: dopamine-2-receptor; D3: dopamine-3-receptor; D4: dopamine-4-receptor; D5: dopamine-5-receptor; BDNF: brain-derived neurotrophic factor; GFAP: glial fibrillary acidic protein; Iba1: ionised calcium binding adaptor molecule 1; IL-1β: interleukin 1-beta; Arg1: aringase-1; SOD1: superoxide dismutase; ADNP: activity dependent neuroprotector homeobox; PACAP: pituitary adenylate cyclase activating polypeptide; VIP: vasoactive intestinal peptide.

## Data Availability

The raw data generated and/or analysed during the current study are available upon reasonable request.

## Supplementary Materials

The following supporting information can be downloaded at: https://www.mdpi.com/article/10.3390/biomedicines10123174/s1.
