# Correction: Thomas Broome, S.; Castorina, A. Systemic Rotenone Administration Causes Extra-Nigral Alterations in C57BL/6 Mice. *Biomedicines* 2022, *10*, 3174

**DOI:** 10.3390/biomedicines13102499

**Published:** 2025-10-14

**Authors:** Sarah Thomas Broome, Alessandro Castorina

**Affiliations:** Laboratory of Cellular and Molecular Neuroscience, School of Life Sciences, Faculty of Science, University of Technology Sydney, Sydney 2007, Australia; sarahthomasbroome@gmail.com

## 1. Error in Figure 1

In the original publication [1], there was a mistake in Figure 1 (panel e) involving an incorrect representative heatmap of mice treated with rotenone (1 mg/kg) for 7 days. The corrected version of Figure 1 appears below.

## 2. Text Correction

According to the suggestion of the Academic Editor, we included the original images as supplementary material. A correction has been made to Section 2.5. Protein Extraction and Western Blot.

When possible, membranes pertaining to the same experimental group were stripped using a mild stripping buffer (1.5% [*w*/*v*] glycine, 1% [*w*/*v*] SDS, and 1% (*v*/*v*) Tween-20 in milliQ H20, pH 2.2) and re-incubated with GAPDH for quantification. Corresponding full blots are available in the Supplementary Material.

A correction has also been made to the Supplementary Material.

The following supporting information can be downloaded at: https://www.mdpi.com/article/10.3390/biomedicines10123174/s1.

The authors state that the scientific conclusions are unaffected. This correction was approved by the Academic Editor. The original publication has also been updated.

## Figures and Tables

**Figure 1:**
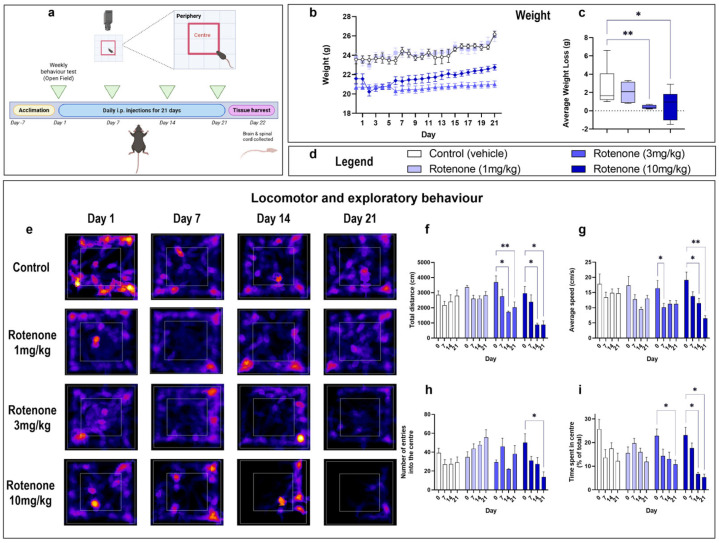
Rotenone impairs locomotor and exploratory behaviour. Experimental timeline for injections and behavioural assessments (**a**). The Open Field Test mice was used to assess locomotor and exploratory behaviour in rotenone-treated mice vs. baseline measurements every 7 days. The centre quadrant was defined as a central square with a surface area that is 25% smaller than the total area (red box), whereas the peripheral area was defined as the surface area between the centre quadrant and the walls of the Open Field box. Mice received an intraperitoneal injection of the indicated treatment daily for 21 days. On day 22, mice were humanely sacrificed and the brain and spinal cord were collected. Mean daily weight per treatment group (**b**) and the average weight loss/gain (**c**) were calculated using mean daily weights of day 1 vs. day 22. Corresponding legends of experimental treatments are shown in (**d**). Representative heat maps from MouBeAt Software that track the movement of mice during the open field test (**e**). Locomotor and behavioural measurements were determined by MouBeAt software. Heat maps use colour to represent time spent in a given area, ranging from blue (short duration) to red/yellow (long duration). Comparisons were made within the same treatment group compared to baseline measurements. An entry into an area was counted when at least 7% of the mouse body had completely entered the area. Total distance travelled (cm) in 5 min (**f**). Average speed reported as cm/s (**g**). Number of entries in centre (**h**). Time spent in centre (s) (**i**). Data are shown as means of n = 6–10 mice per group. * *p* < 0.05 or ** *p* < 0.01 as determined by one-way ANOVA followed by Dunnett’s post hoc test (weight change) or two-way repeated measures ANOVA followed by Tukey post hoc test (behavioural measurements).
