# Supplementary material for: Systemic Rotenone Administration Causes Extra-Nigral Alterations in C57BL/6 Mice
Source: Biomedicines. 2022 Dec 7;10(12):3174. doi: 10.3390/biomedicines10123174 (PMC9775048; doi:10.3390/biomedicines10123174)

# Raw western blot images – Figure 2 (TH & DAT)

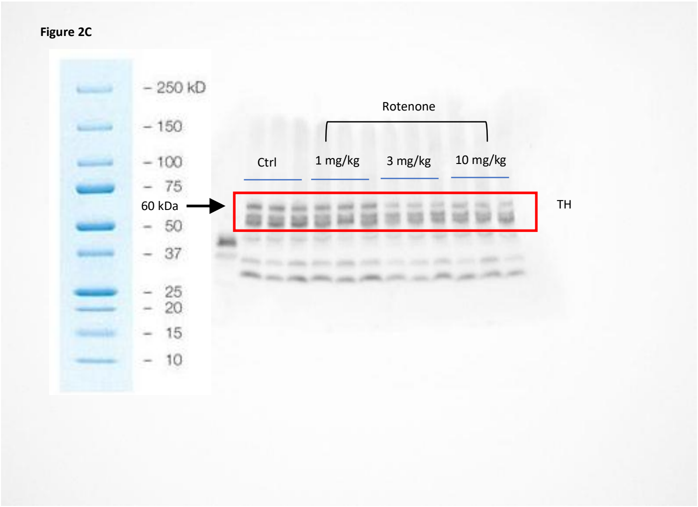


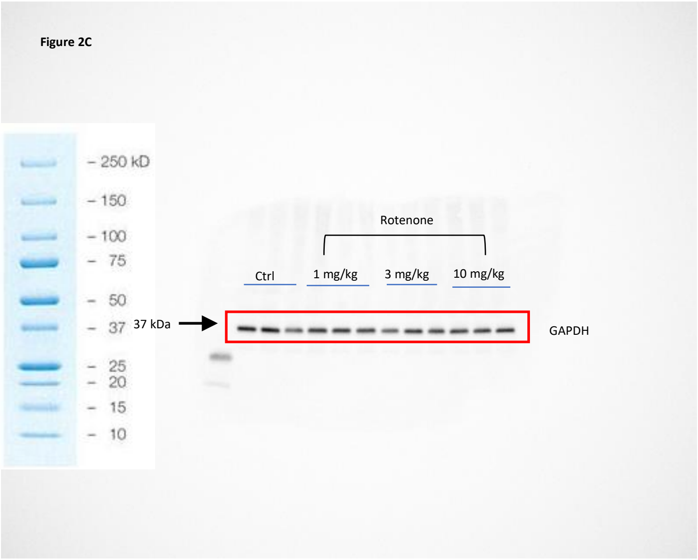

**Figure 2F**

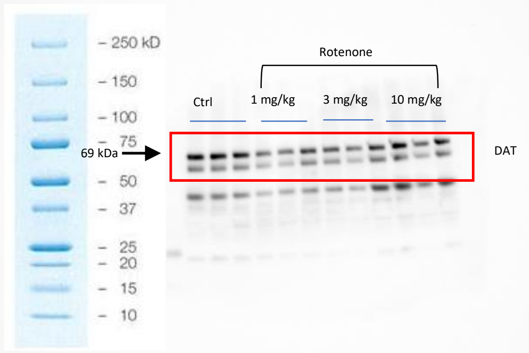


**Figure 2F**

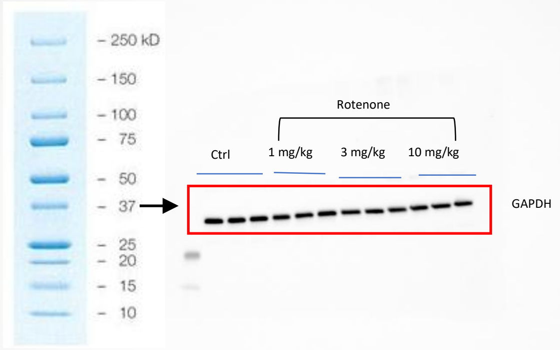

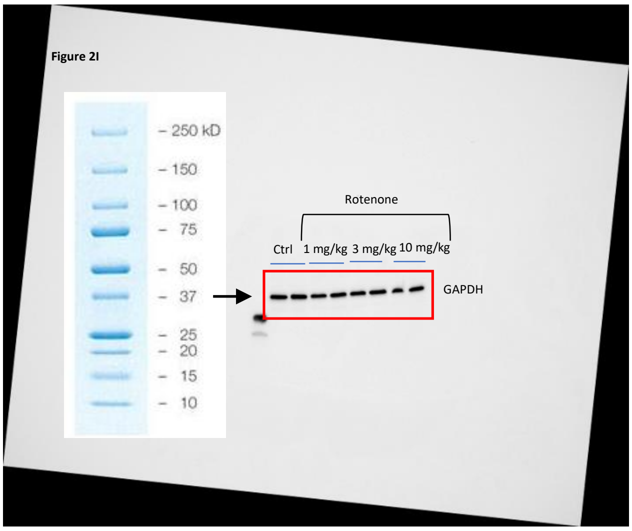

Figure 2I
250 kD
150
100
75
50
37
25
20
15
10
Rotenone
Ctrl
1 mg/kg
3 mg/kg
10 mg/kg
GAPDH


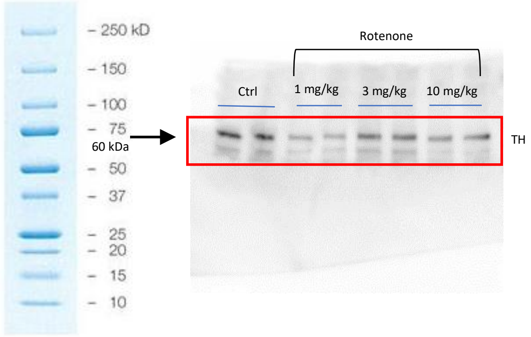

250 kD
150
100
75
60 kDa
50
37
25
20
15
10
Rotenone
Ctrl
1 mg/kg
3 mg/kg
10 mg/kg
TH


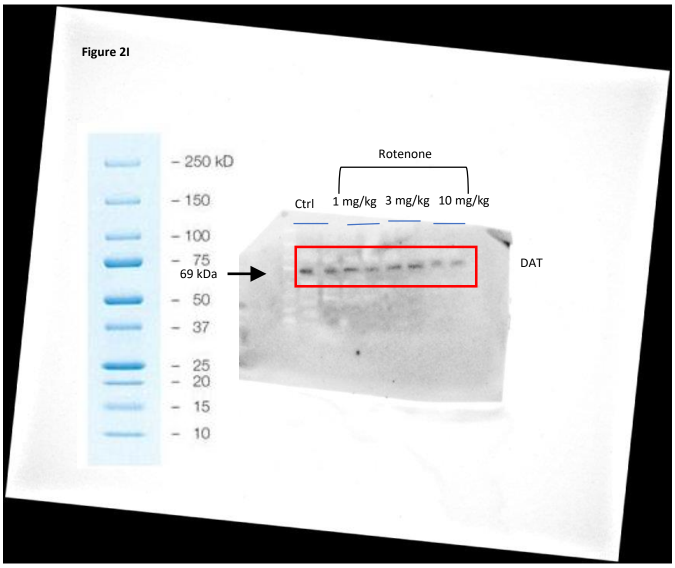

Figure 2I
250 kD
150
100
75
69 kDa
50
37
25
20
15
10
Rotenone
Ctrl
1 mg/kg
3 mg/kg
10 mg/kg
DAT

# Raw western blot images – Figure 3 (BDNF)

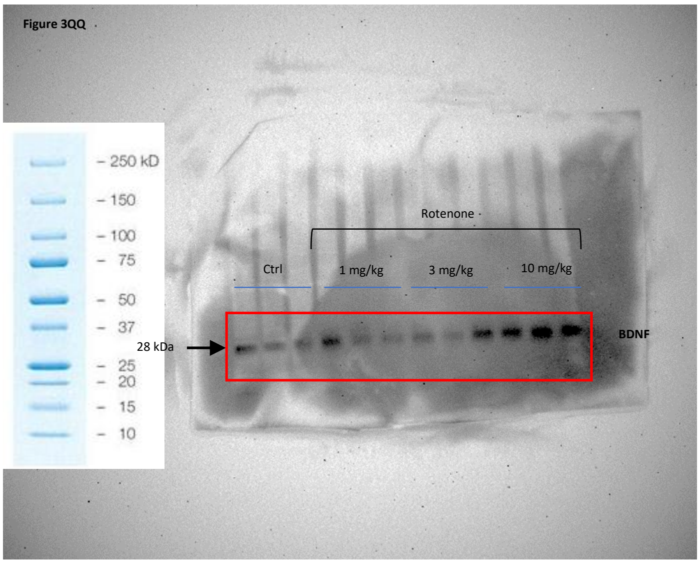


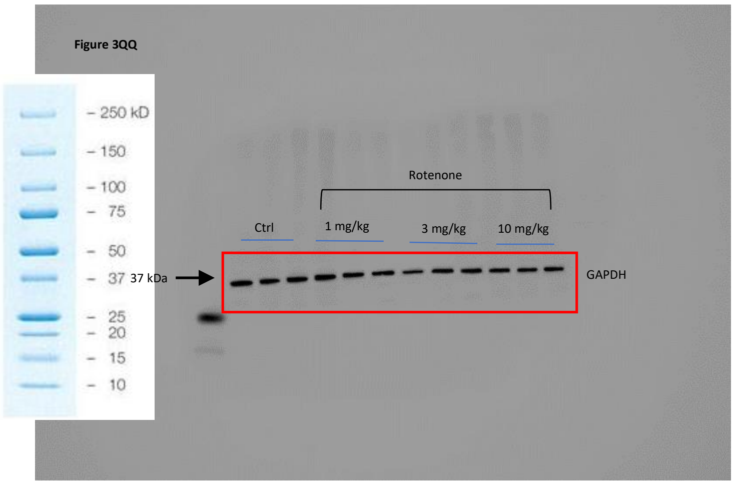

# Raw Western blot images – Figure 4 (OPA1 & SOD1)

## Figure 4C – Midbrain

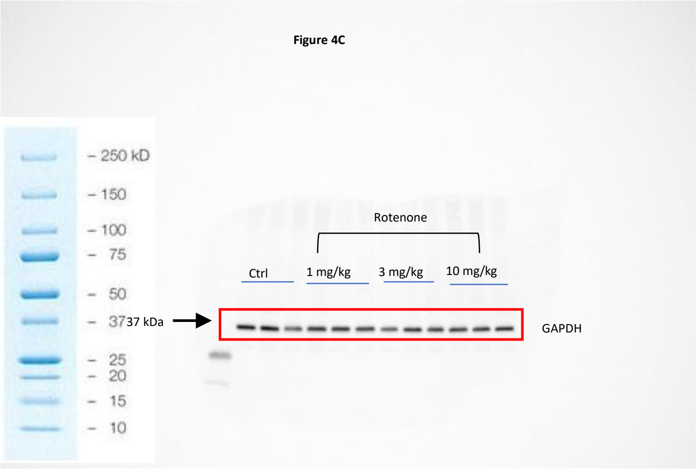


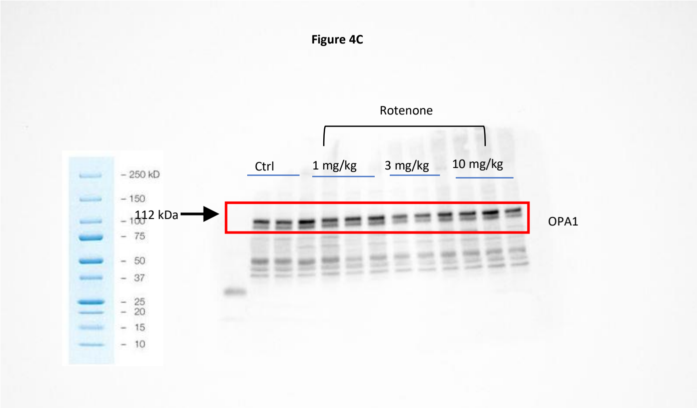


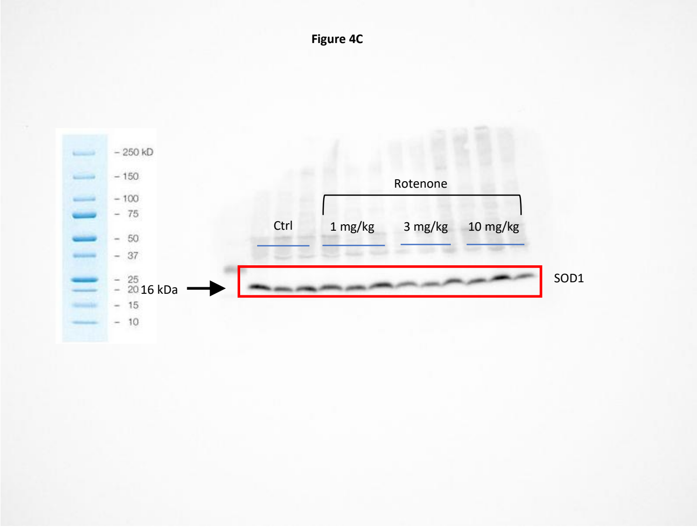

# Figure 4H – Striatum

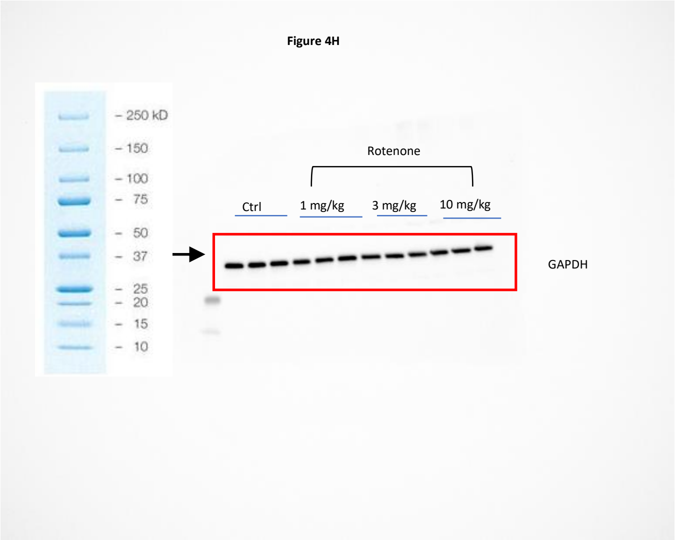


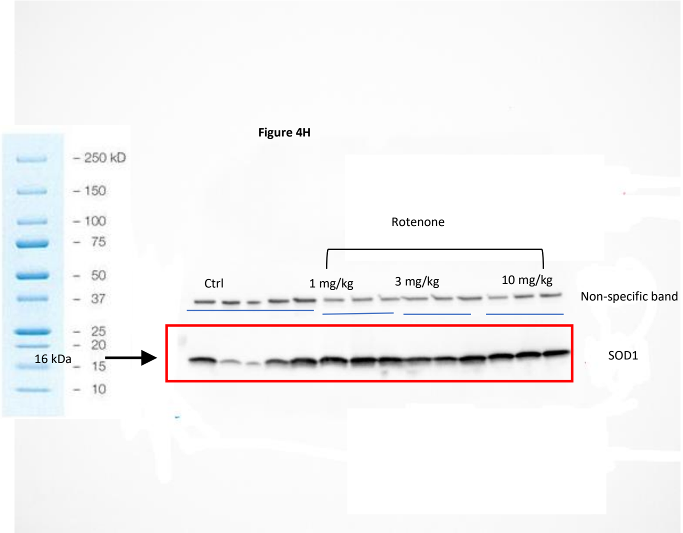


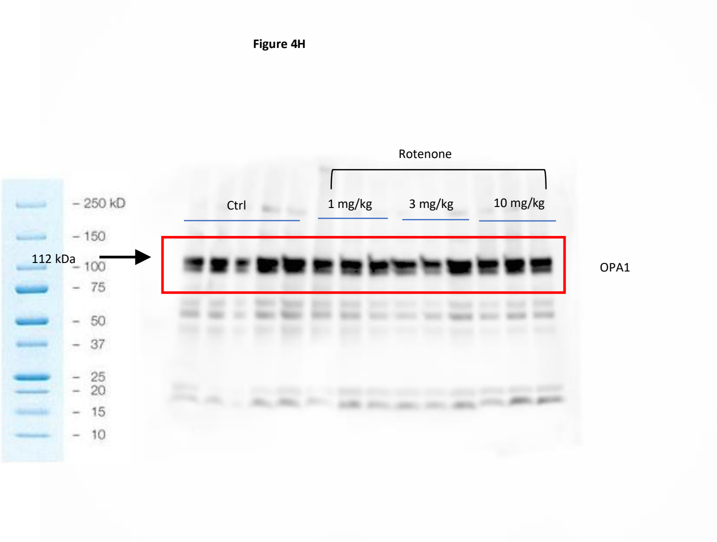

## Figure 4M – Prefrontal cortex

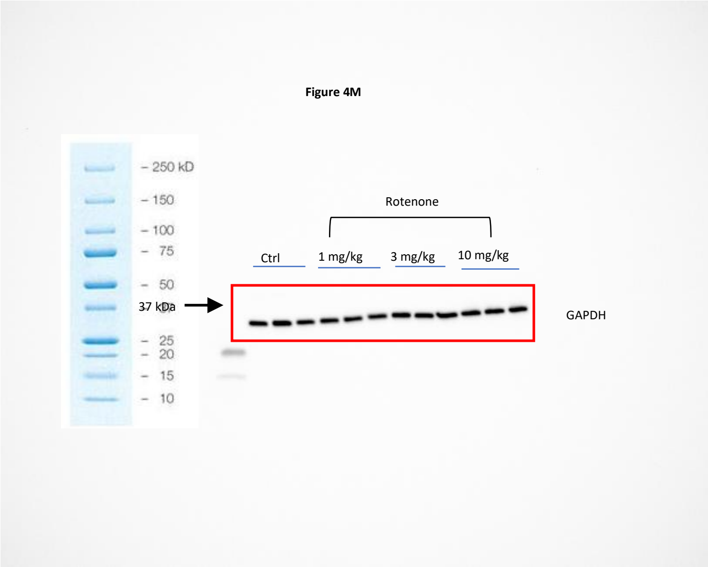


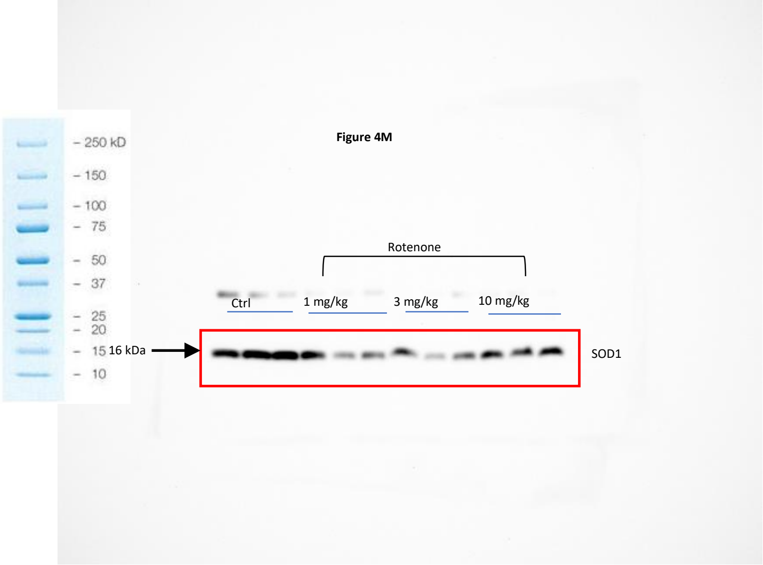


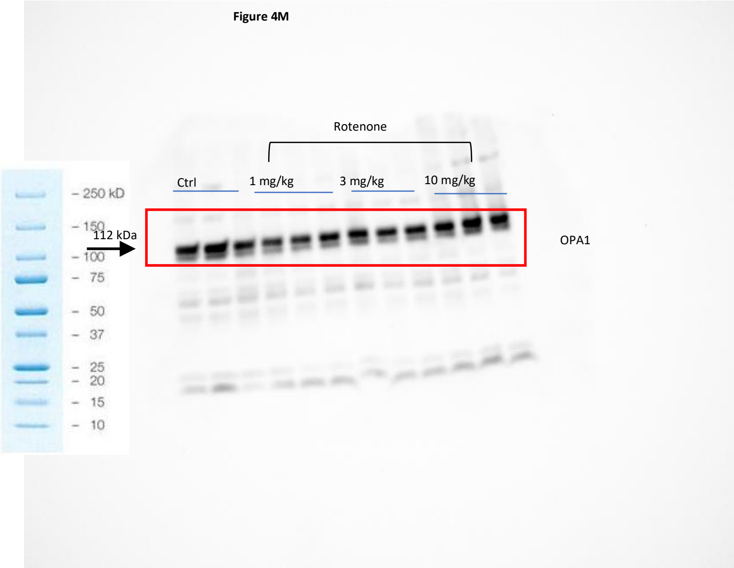

# Figure 4R – Amygdala

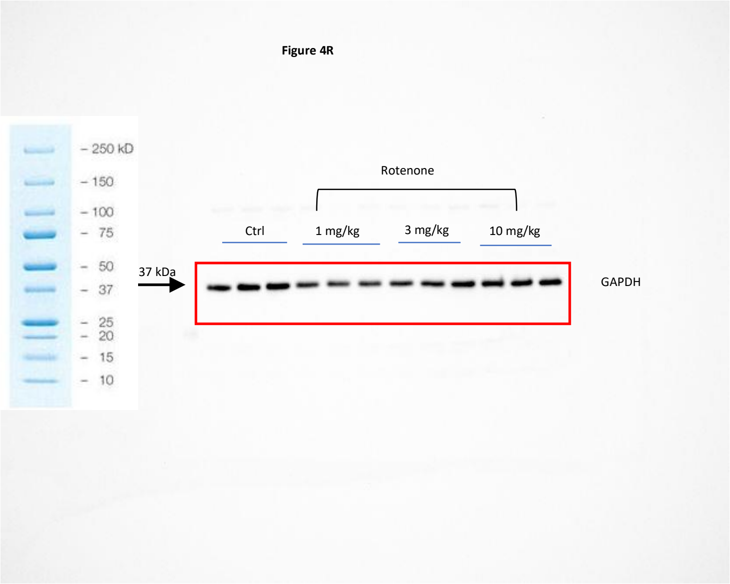


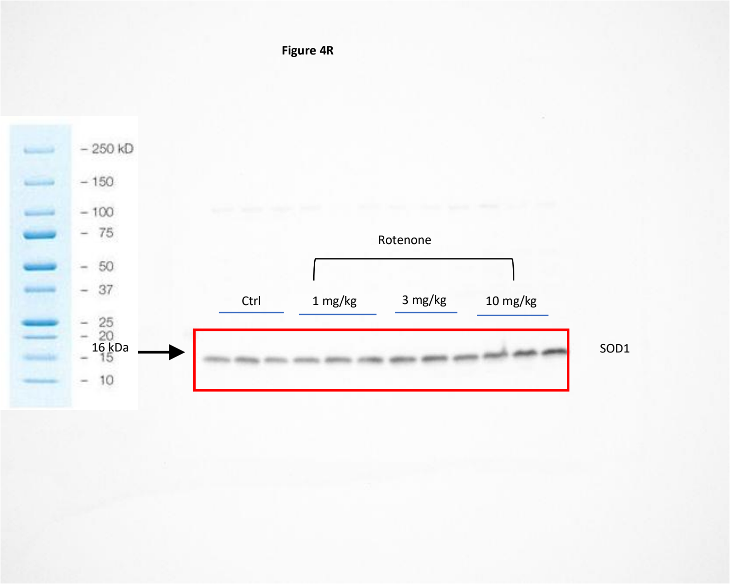


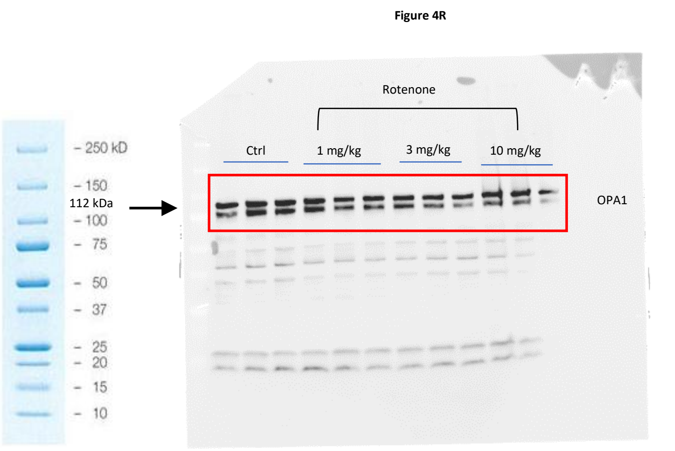

# Figure 4W – Hippocampus

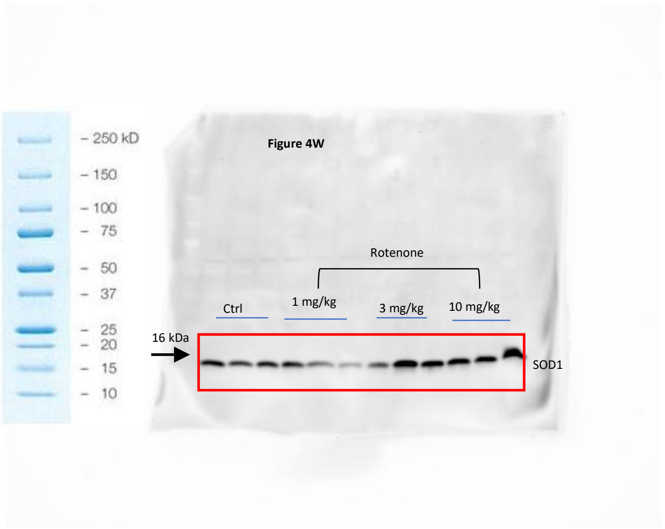


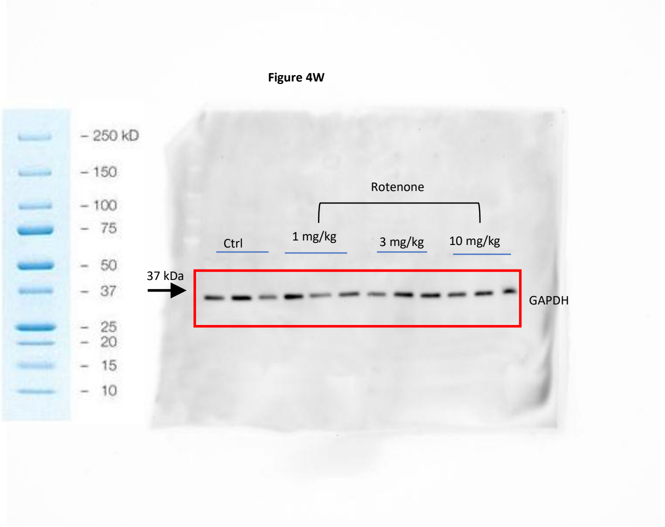


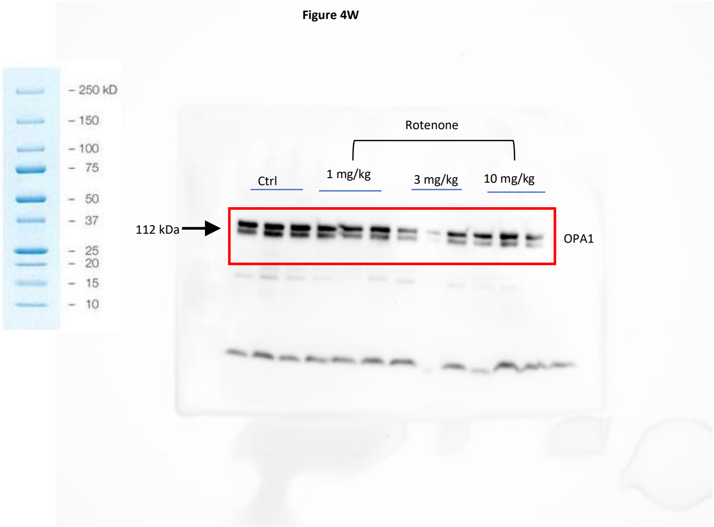

# Figure 4BB – Spinal cord

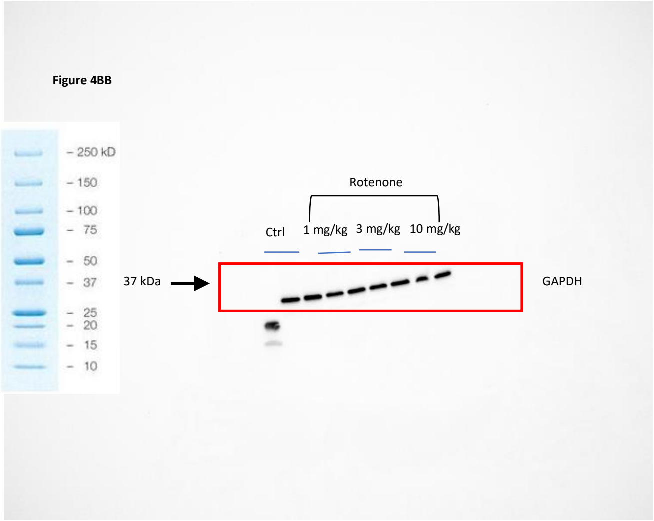


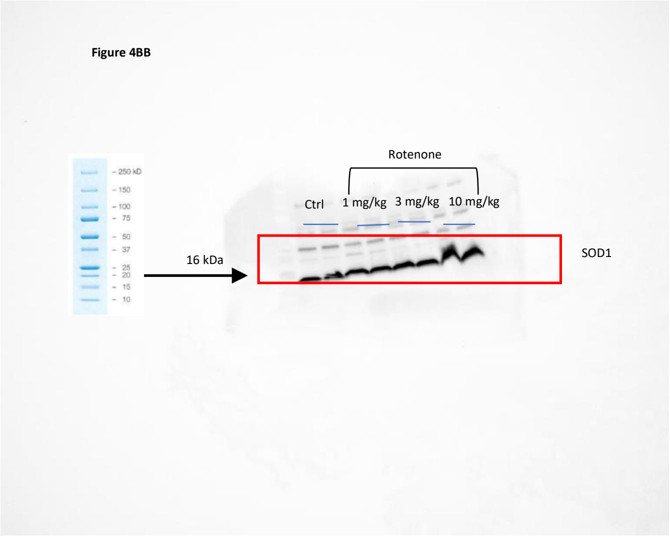


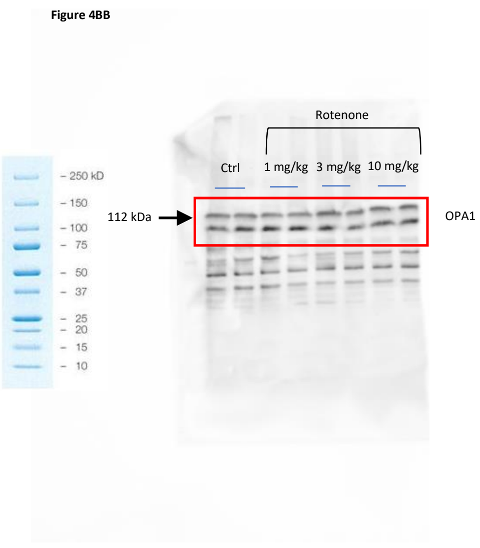

# Raw western blot images – Figure 5 (CD11B & GFAP)

## Figure 5E – Midbrain

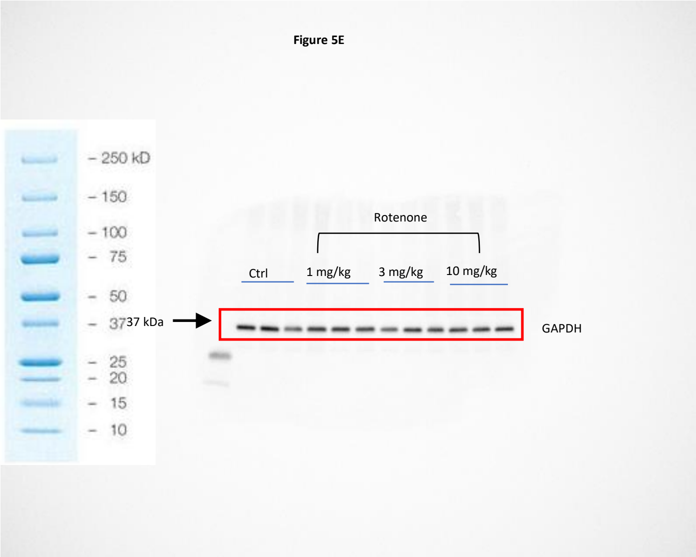


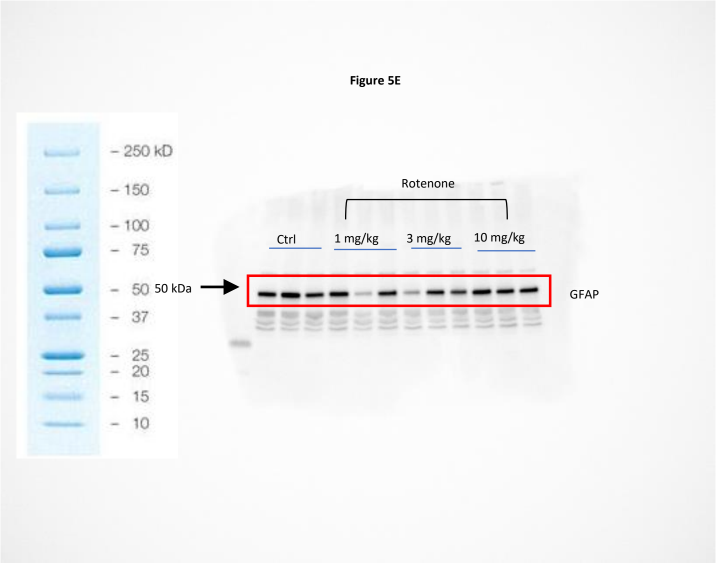


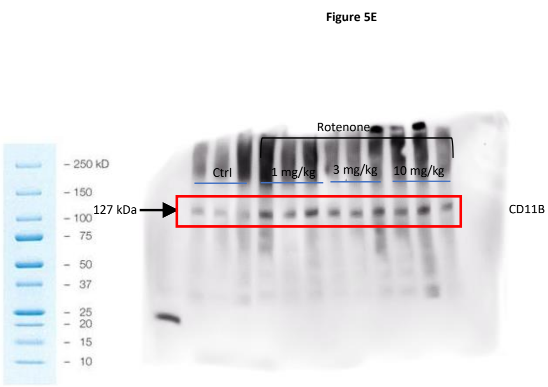

# Figure 5L – Striatum

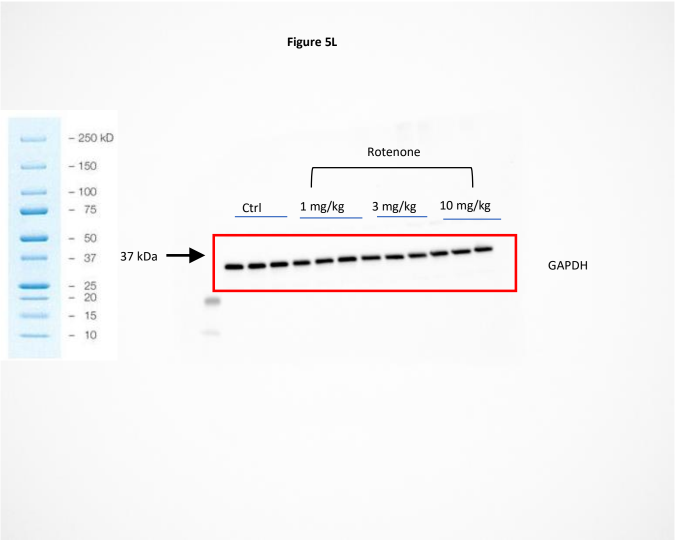


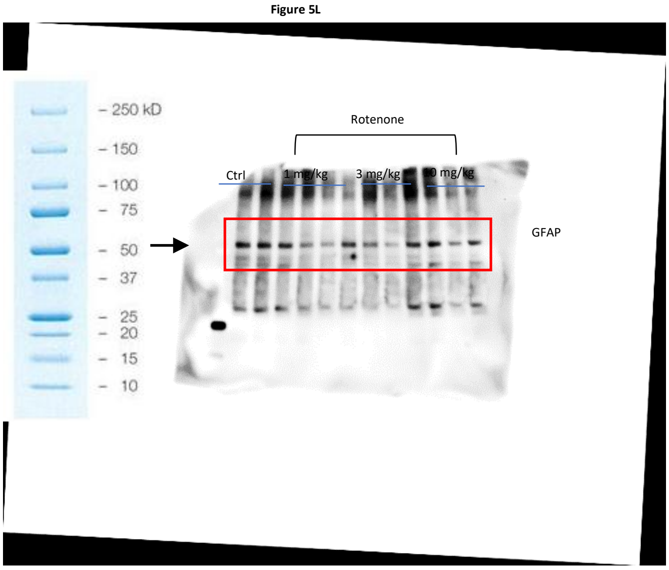


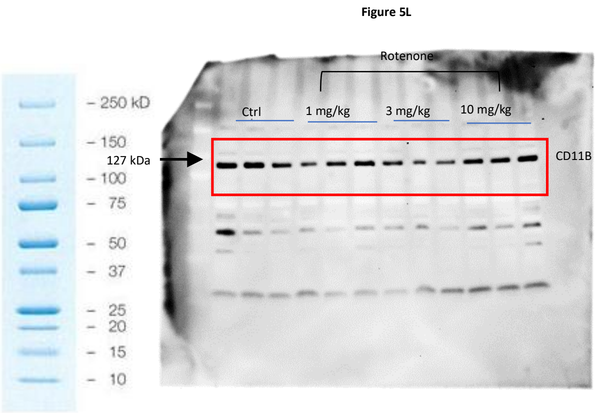

# Figure 5S – Prefrontal cortex

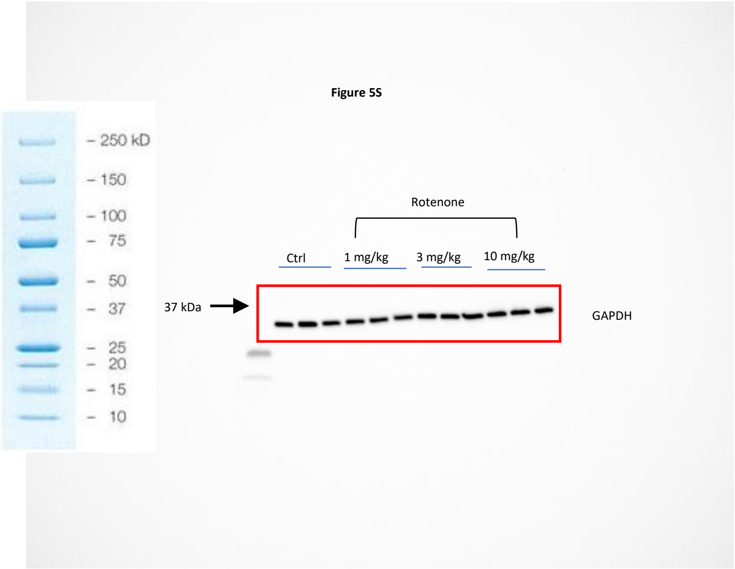


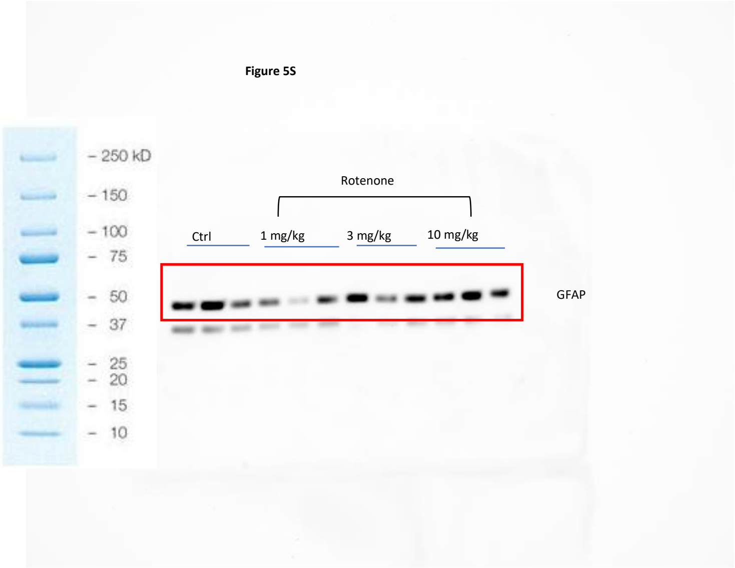


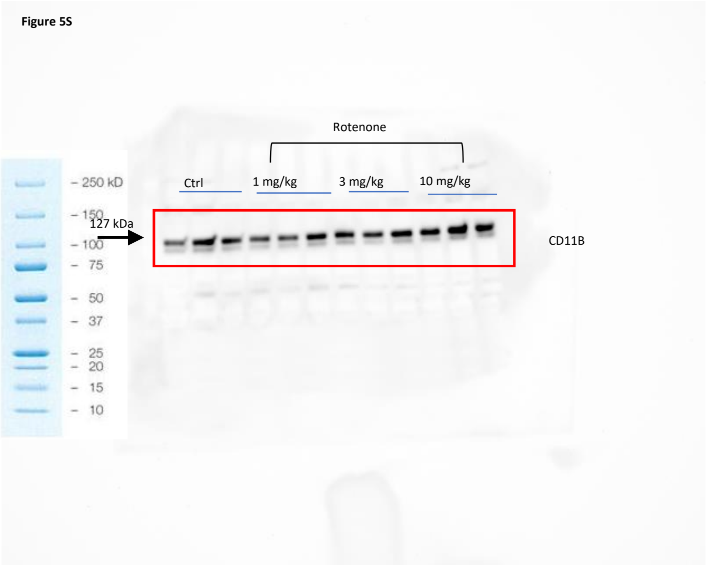

## Figure 5Z – Amygdala

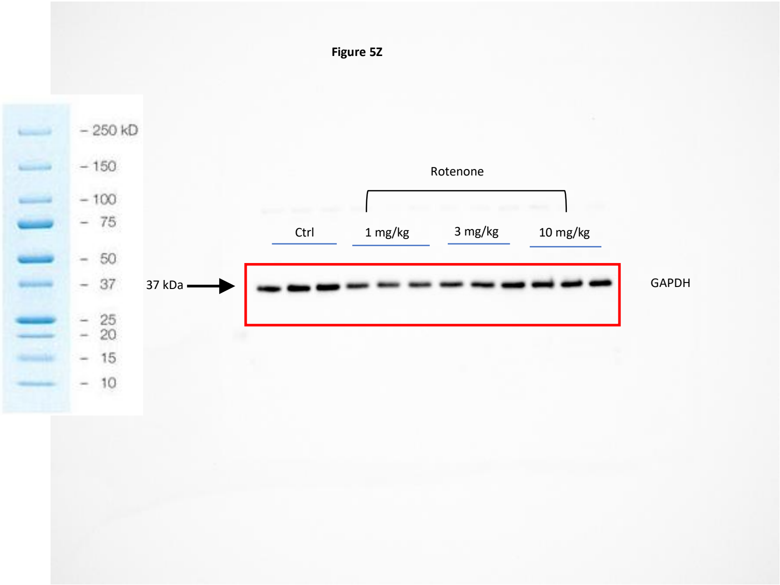


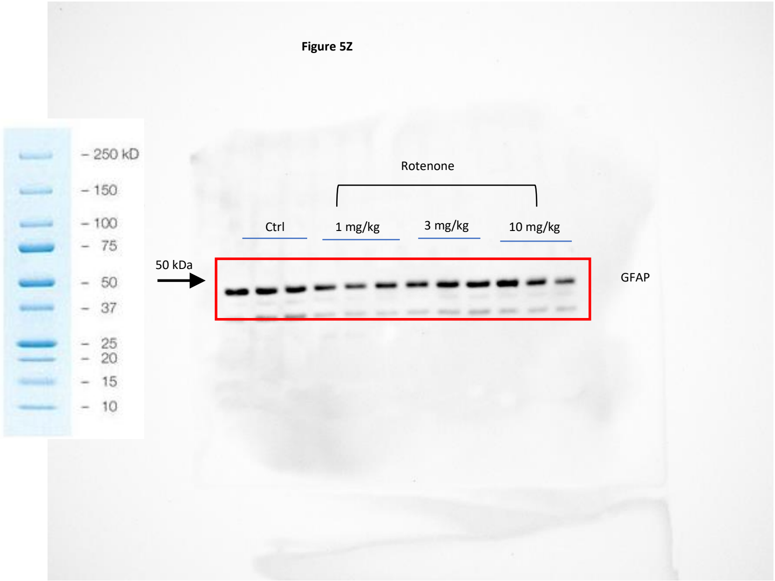


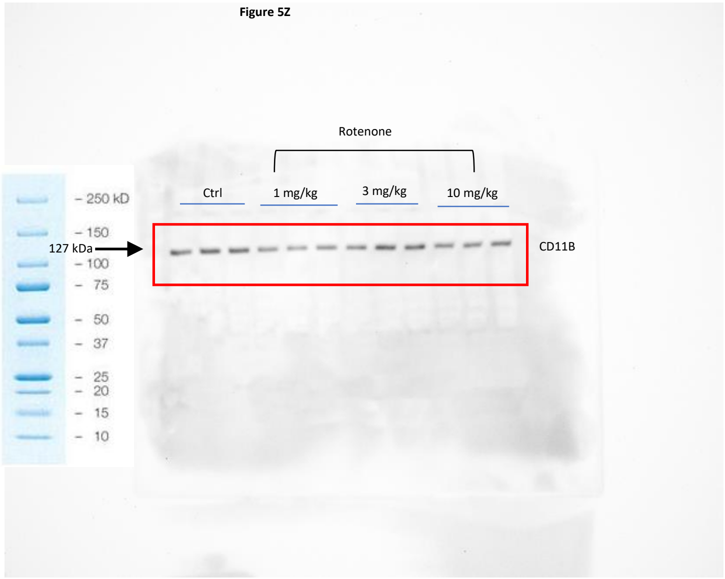

# Figure 5gg – Hippocampus

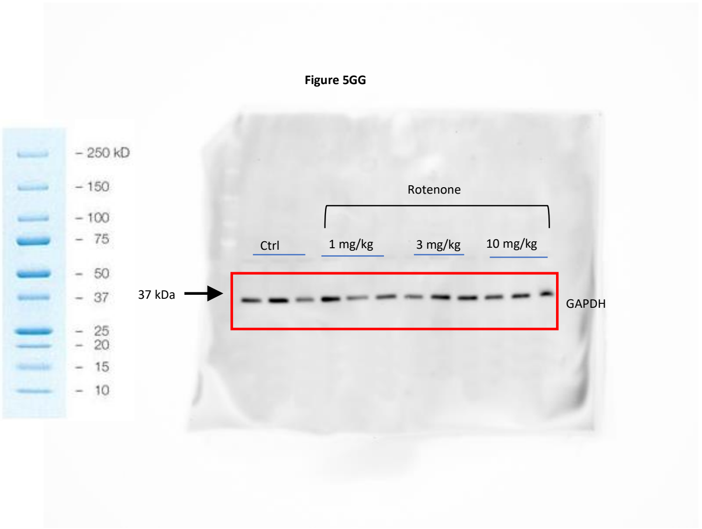


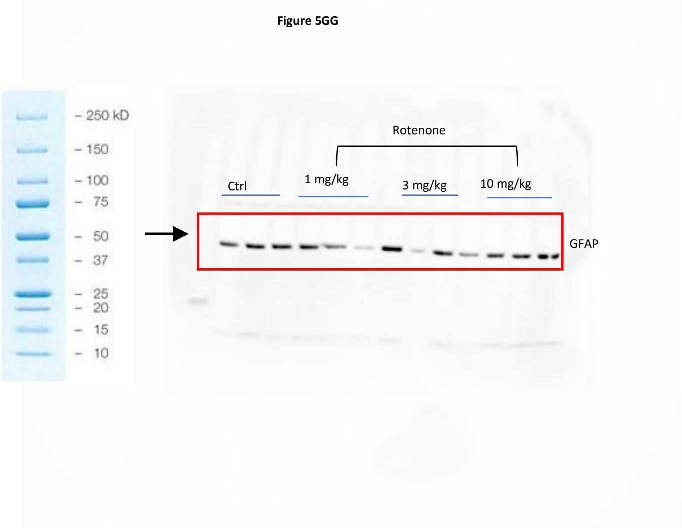


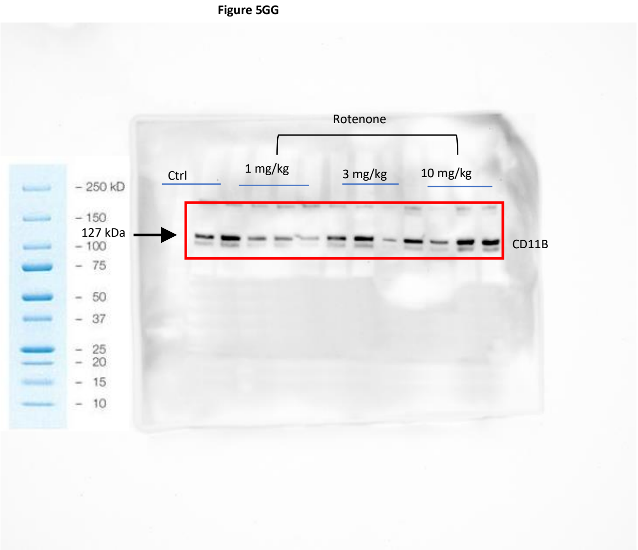

## Figure 5nn – Spinal cord

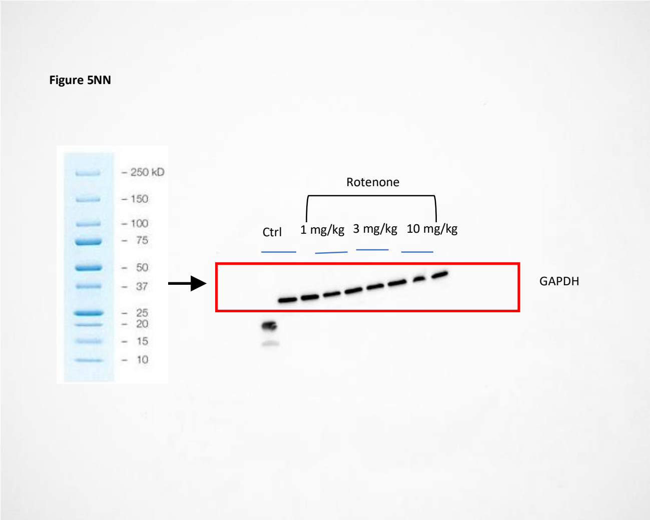


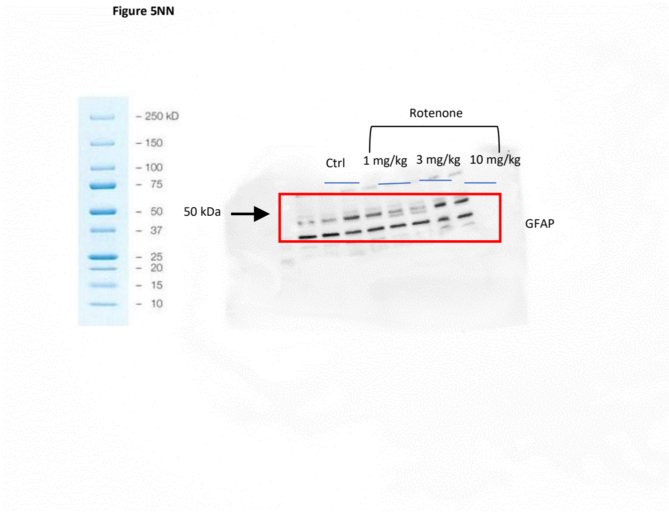


Figure 5NN

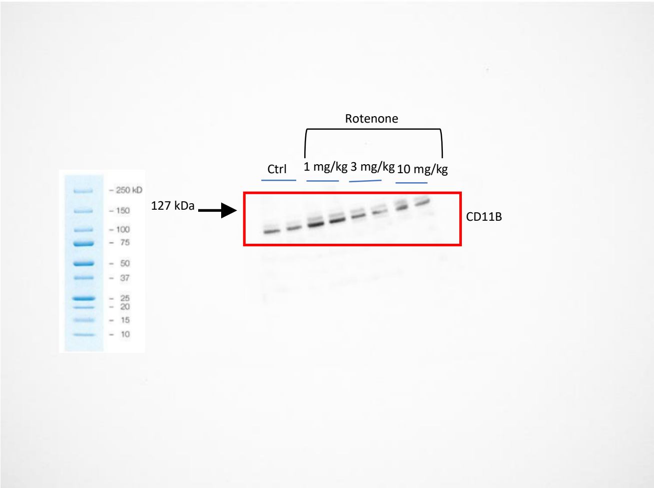

## Raw western blot images – Figure 6 (PACAP & VIP)

## Figure 6D – Midbrain

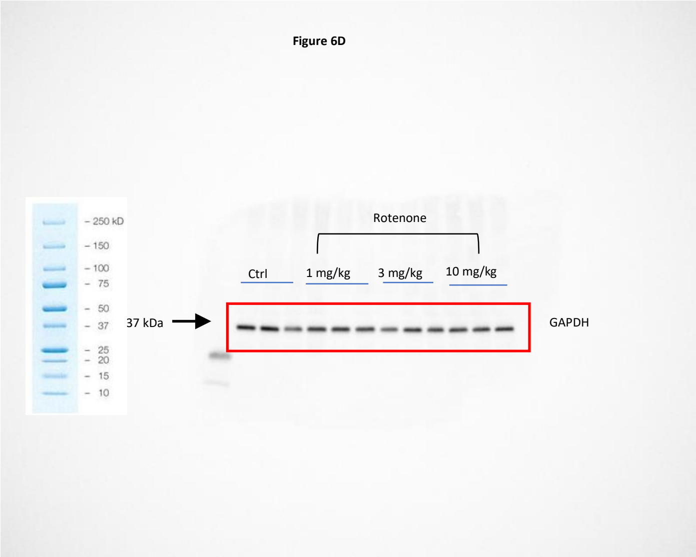


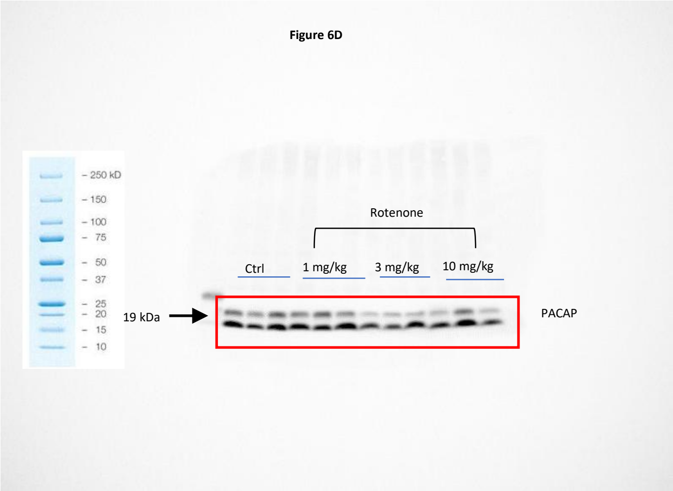


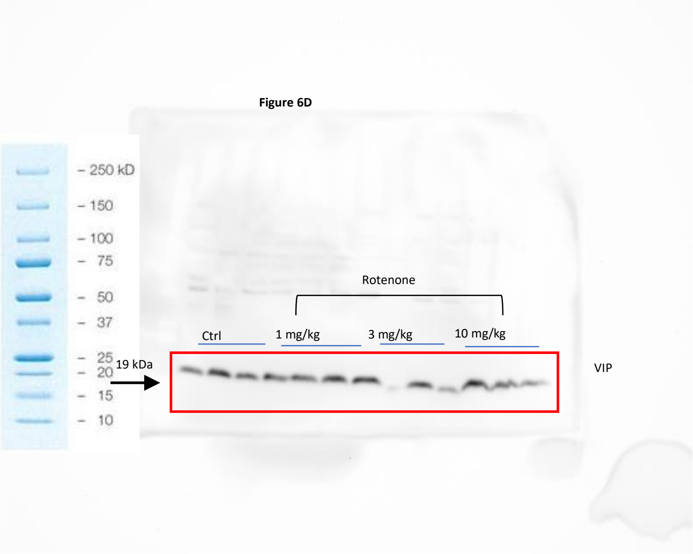

# Figure 6J – Striatum

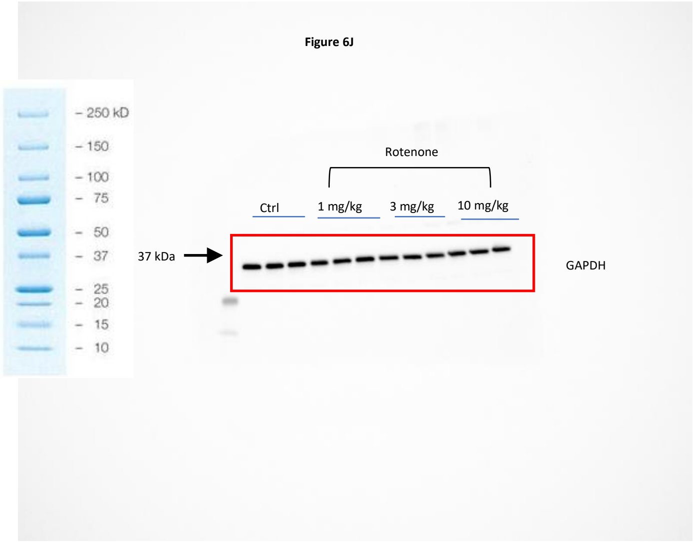


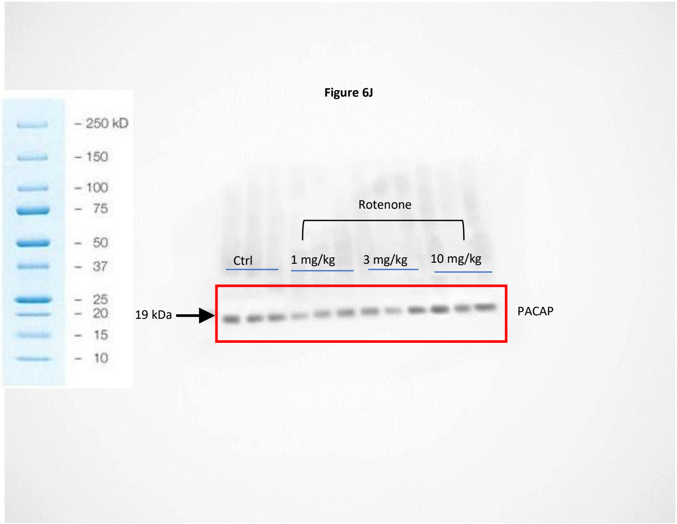


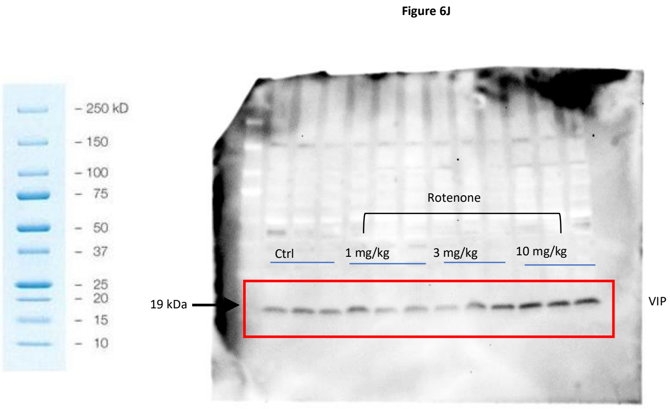

# Figure 6P – Prefrontal cortex

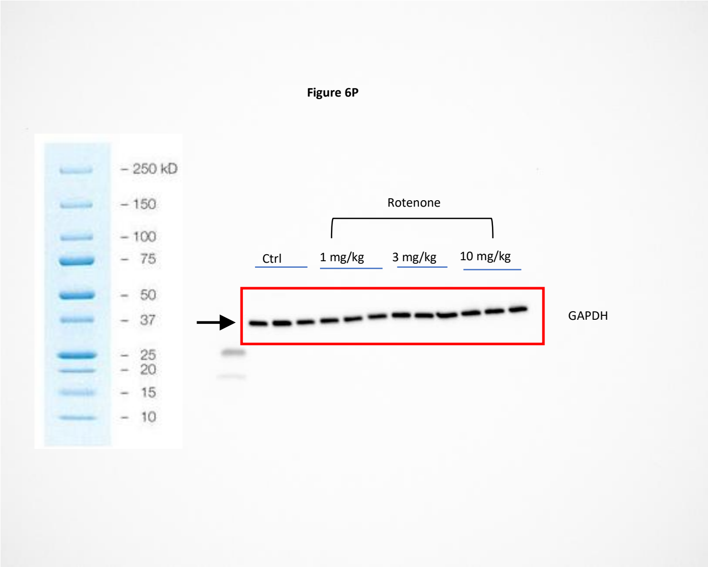


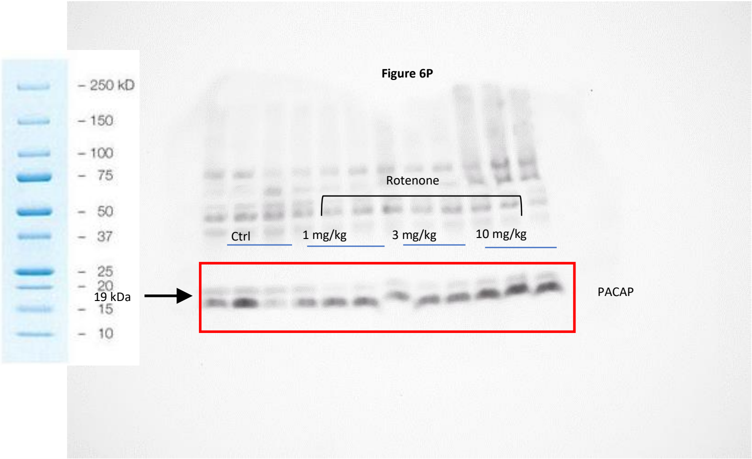


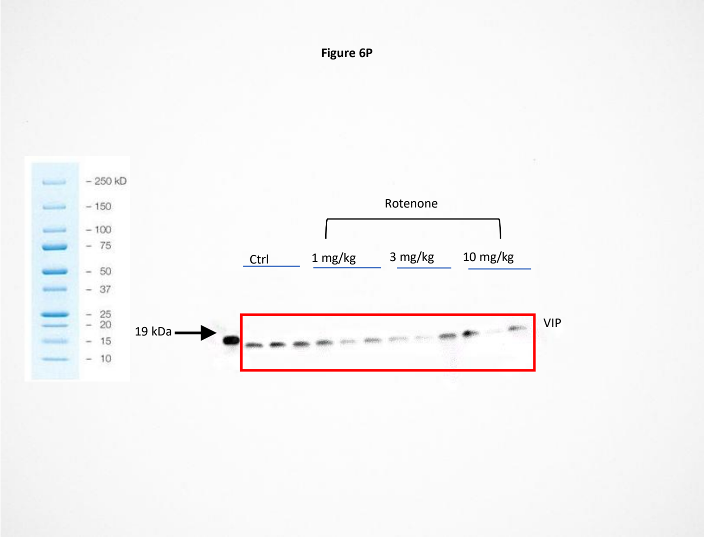

## Figure 6V – Amygdala

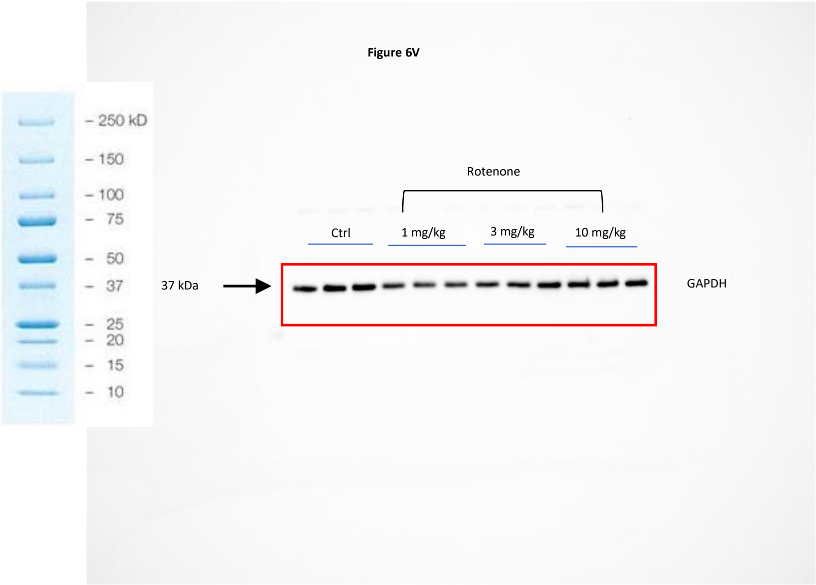


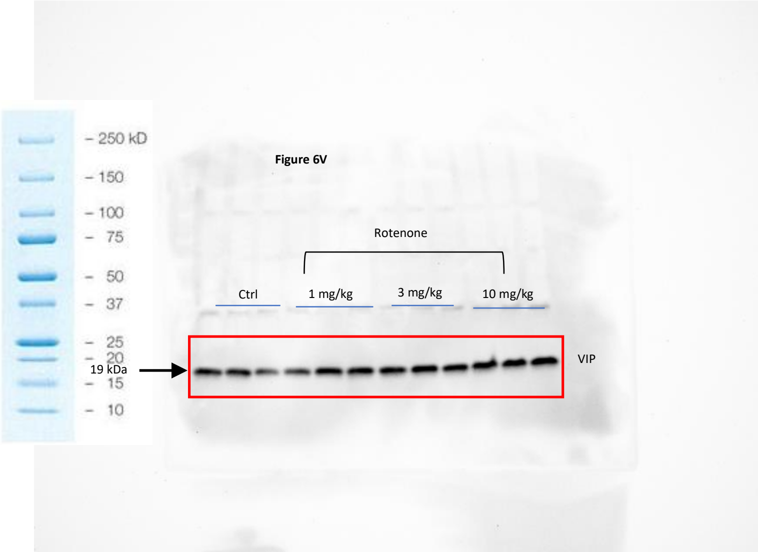


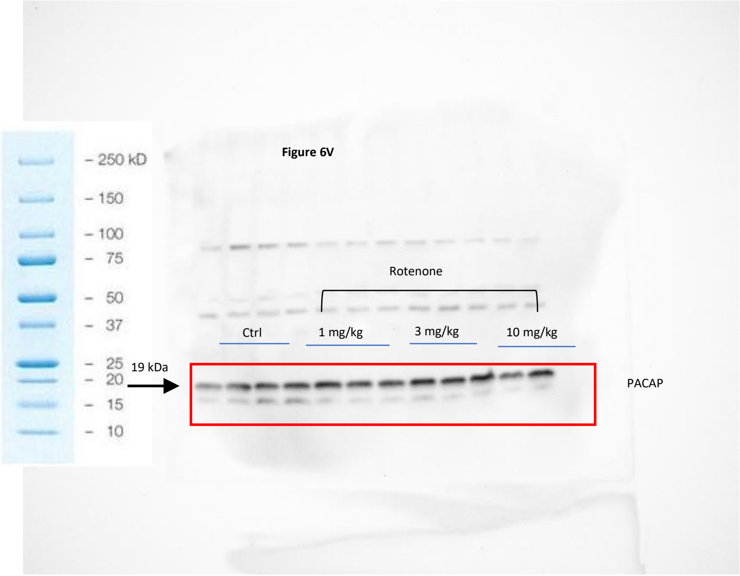

# Figure 6BB – Hippocampus

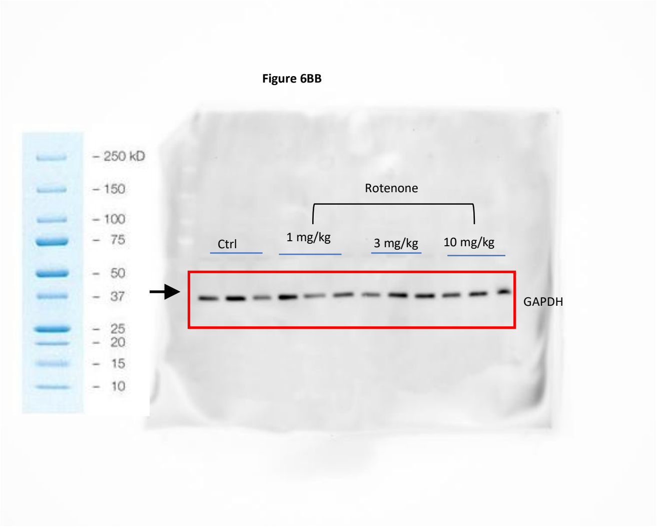


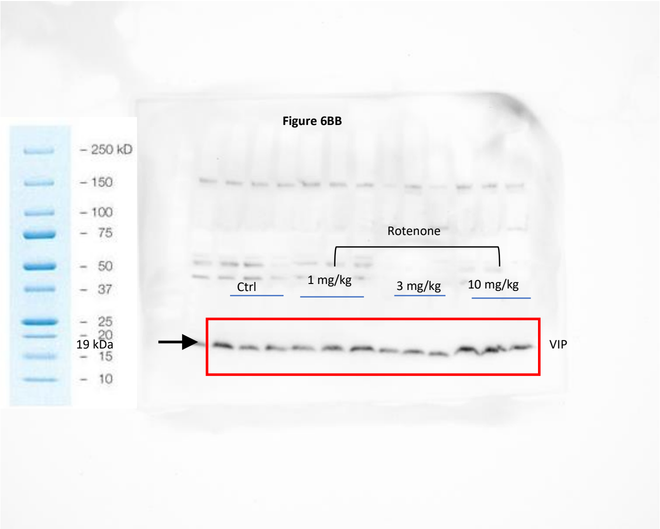


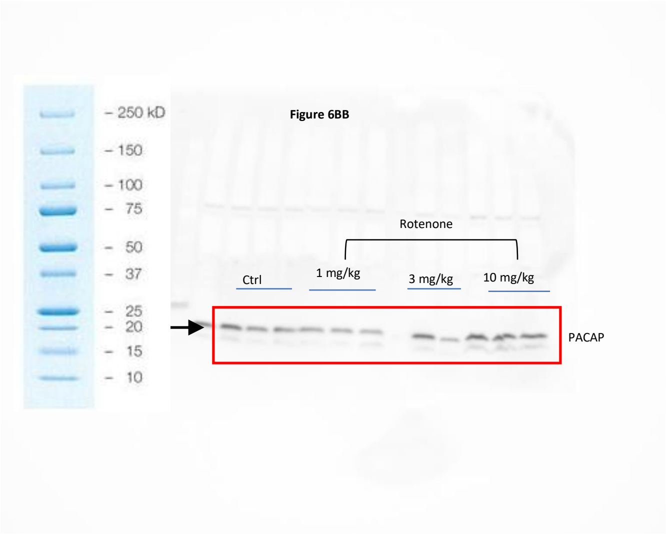

## Figure 6HH – Spinal cord

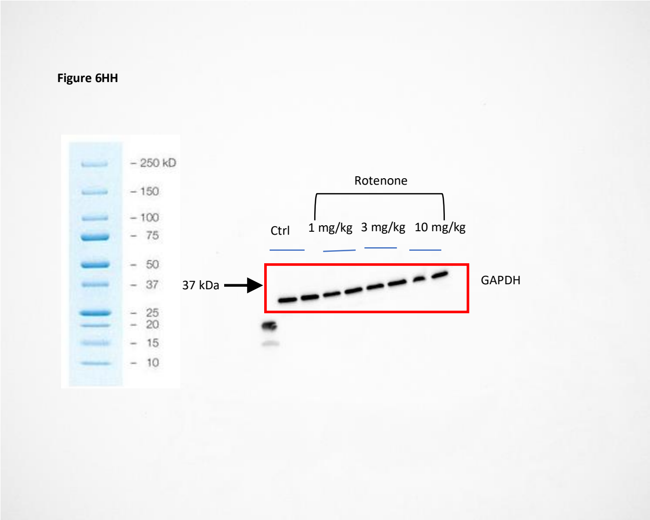


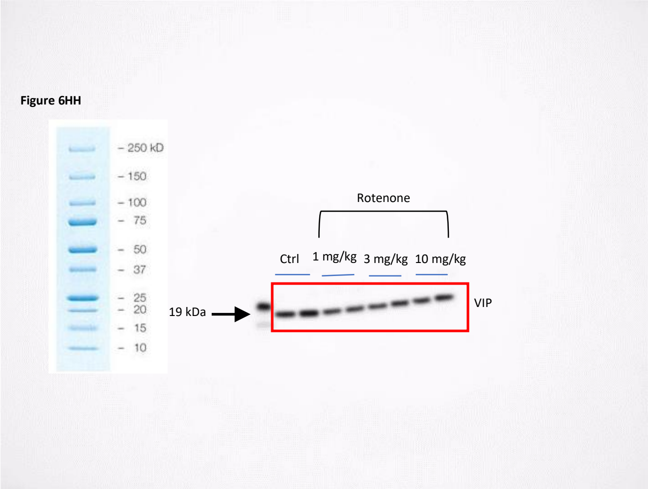


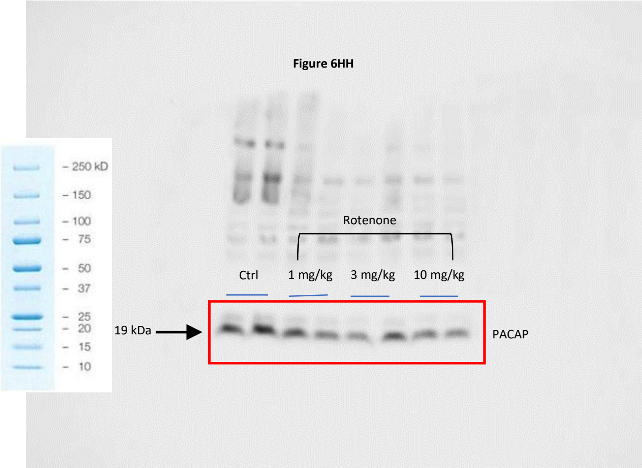

Supplement: Supplementary file 1 [file biomedicines-10-03174-s001.zip › biomedicines-2004289-supplementary.pdf]
